# Reconfigurable Intelligent Surface-Aided Cooperative NOMA with *p-CSI* Fading Channel toward 6G-Based IoT System

**DOI:** 10.3390/s22197664

**Published:** 2022-10-09

**Authors:** Hsing-Chung Chen, Agung Mulyo Widodo, Jerry Chun-Wei Lin, Chien-Erh Weng

**Affiliations:** 1 Department of Computer Science and Information Engineering, Asia University, Taichung City 413305, Taiwan; 2 Department of Medical Research, China Medical University Hospital, China Medical University, Taichung City 404327, Taiwan; 3 Department of Computer Science, Universitas Esa Unggul, Jakarta 11510, Indonesia; 4 Department of Computer Science, Electronic Engineering and Mathematical Science, Western Norway University of Applied Sciences, 5063 Bergen, Norway; 5 Department of Microelectronics Engineering, National Kaohsiung University of Science and Technology, Kaohsiung City 81157, Taiwan

**Keywords:** reconfigurable intelligent surfaces (RIS), non-orthogonal multiple access (NOMA), outage probability, ergodic capacity, perfect statistical channel state information (*p*-CSI)

## Abstract

Addressing the challenges of internet-based 5G technology, namely increasing density through micro-cell systems, frequency spectrum, and reducing resource costs, is needed to meet the use of IoT-based 6G technology with the goal of high-speed, high-capacity, and low-latency communication. In this research, we considered the coverage performance and ergodic capacity of the Reconfigurable Intelligent Surface (RIS)-aided cooperative nonorthogonal multiple-access network (NOMA) of an IoT system. This enables the upgrading of 5G- toward 6G-technology-based IoT systems. We developed a closest-form formula of near and far user coverage probabilities as a function of perfect channel statistical information (*p*-CSI) using only a single-input single-output (SISO) system with a finite number of RIS elements under the Nakagami-*m* fading channel. We also define ergodic capacity as a simple upper limit by simplifying the use of symbolic functions and it could be used for a sustained period. The simulation findings suggest that RIS-assisted NOMA has a reduced risk of outage than standard NOMA. All of the derived closed-form formulas agree with Monte Carlo simulations, indicating that the distant user’s coverage probability outperforms the nearby user. The bigger the number of RIS parts, however, the greater the chance of coverage. They also disclose the scaling law of the number of phase shifts at the RIS-aided NOMA based on the asymptotic analysis and the upper bound on channel capacity. In both arbitrary and optimum phase shifts, the distant user’s ergodic capacity outperforms the near user.

## 1. Introduction

The advancement of the modern Internet has spurred a 1000-fold rise in data traffic for 5G. Consequently, spectral efficiency has become one of the most crucial factors in addressing such huge data flows. Furthermore, due to the hurried expansion of the Internet of Things (IoT), 5G will need to support considerable connectivity of people and/or appliances in order to meet the demand for low latency, inexpensive appliances, and a range of services. Multiple IoT appliances typically transmit data to a coordinator while this coordinator and other coordinators communicate with a server [1,2]. However, this explosion in demand requires large resources because it requires 5G network infrastructure involving mm-Wave frequencies to cover only short distances [3], thus requiring an ultra-dense network. For this reason, 5G small cells will be used in a much wider range of scenarios than in the past, and their form factors and architectures will vary widely. This results in the additional costs of spectrum purchasing, network configuration, network testing, management, and maintenance.

Depending on the location of each 5G small cell, installing a fiber optic network is not always practical. As a result, 5G must incorporate wireless network growth. The potential of 5G can provide bandwidth at frequencies higher than six gigahertz, allowing for the networking required by consumers with devices capable of higher data rates. The high-frequency spectrum, on the other hand, is limited. Thus, multiple tiny cells are required to cover a large region. The signals could be blocked by trees, buildings, and other objects. Therefore, it necessitates the use of cell towers to avoid signal loss. In addition, the application of small-cell 5G also needs to be considered in special locations, such as in tunnels both below and above the ground. In particular, the small-cell 5G is used for high-speed rail communication systems.

The multiple-input, multiple-output (MIMO) technology has been proved to increase 5G connectivity capacity while also addressing signal-route issues. Massive MIMO uses a single frequency to service numerous mobile devices in a congested region. A huge MIMO network can survive signal interference by using more antennas and as potential alternatives to overcome 5G difficulties so far with Ref. [1]. However, the sight line will still be a 5G issue when high frequencies are involved, so more base stations on rooftops or towers are needed. This creates new problems in terms of providing new spaces on the roof top and in tower locations.

In addition, NOMA as a 5G basis still requires a reduction in energy requirements in the amplify and forward (AF) process. Therefore, a technology other than 5G is needed that is able to reduce the weakness of NOMA. This is what motivated the authors to develop NOMA as the basis for 5G system in order to be able to achieve the principles of the 6G system by implementing RIS-aided NOMA.

Technological upgrades are needed to encounter the goals of high-speed, high-capacity, and low-latency communication but also to overcome the challenges of 5G technology (density through small-cell systems, frequency spectrum, and reduction in resources and investment costs) called for by the 6G-technology-based Internet of Things (IoT).

To support the achievement of this goal, we propose an IoT system that combines intelligent multi-antennas, called Reconfigurable Intelligent Surfaces (RIS), with NOMA technology applied to cooperative networks.

However, very little research has been conducted to assess analytical performance, and the number of outcomes is quite limited. The authors used the RIS-assisted NOMA network system with Nakagami-*m* as the fading channel model to investigate outage performance, which is a measure of a network system’s coverage. Aside from that, the authors look at the channels’ ergodic capacity for both nearby and distant users. The main contribution of this paper is stated as follows:

- This study compares the performance of the RIS-aided NOMA-based IoT system to that of a traditional NOMA system with two users (nearby users and distant users) and no direct link to the operator base.
- This study derives the closest expression of outage probability for each condition using the RIS-aided wireless system as reflectors and relays. In addition, this study also aims to discover the performance of the outage probability and ergodic capacity at various SNR values for nearby users and distant users.
- The goal of this research is to show that the outage probability and the ergodic capacity in RIS-assisted NOMA wireless networks have better performance than in a traditional NOMA wireless network. Our research shows that RIS-aided NOMA-based IoT systems could replace traditional NOMA relay systems. A mathematical model relating to the outage probability and the ergodic capacity performance of each receiver, both near and far from the base station, have been constructed for this purpose.

This paper is organized as follows. Section 2 explains the system model used in this research. Section 3 explains how the system model is used, and the closest expression of the transmitted signal by base station and received signals by each of the users. Section 4 explains the performance analysis through a mathematical approach in order to obtain the closest expression of the outage performance plotted in the Cartesian plane. Section 5 explains the performance analysis through a mathematical approach in order to obtain the closest expression of the ergodic capacity plotted in the Cartesian plane of each of the users. The plot results of the equations derived are explained in Section 6, which is followed by a discussion. Finally, in Section 5, the study’s conclusion is presented.

## 2. Related Works

To properly utilize previous data available in NOMA systems [4], a cooperative NOMA transmission strategy is presented. An innovative framework, particularly the preface sequence, allows the CCR in a station (STA) to quickly synchronize with an access point (AP) and the PCR to be immediately activated while an AP desires to broadcast data to an STA [5]. Utilizing consecutive recognition at the receivers necessitates those users with valuable channel conditions to decode the messages for those with deteriorating conditions. Because of this, these users can act as relays to help users with weak base station connections receive better reception. The key benefit of NOMA is that it explores the additional power domain to expand the number of users that may be supported users that are recognized specifically by their channel situations. Near users are persons who have excellent channel situations, whereas far users are those who do not. Users are given less authority for the sake of fairness. For multi-user detection, the transmitter combines signals with different levels of power, and each user is subjected to sequential interference cancellation (SIC) by the receiver [6,7,8]. Due to the nonorthogonal resource allocation, NOMA can handle more users, making it a possible candidate for meeting the 5G need of immense connectivity at the cost of a manageable increase in receiver complexity owing to SIC [6].

RIS have been proposed as a potential replacement for phased arrays [9,10,11,12,13,14]. In contrast to traditional phased arrays, almost all of the components that make up RIS are low-power, numerous, and nearly passive. Each of these elements can electronically control the phase of electromagnetic waves that have unusual properties, such as negative refraction, perfect absorption, and unusual reflection [11,15]. Furthermore, the spatial feeding mechanism of RIS eliminates the high-power loss generated by phased arrays’ bulky feeding networks. As a result, power usage and hardware costs are both greatly decreased with RIS. It might be necessary to use more antenna elements, nevertheless, to obtain the same antenna gain as conventional phased arrays. The Nakagami-*m* fading channel is constructed with several types of channels, with Gaussian channel and the Rayleigh fading channel serving as special cases. The performance of NOMA-based AF relay networks was examined in [16], and it was discovered that NOMA outperforms orthogonal multiple access (OMA) in terms of outage probability and ergodic summation value. Over the Nakagami-*m* fading channel, it also delivers superior spectral efficiency and user fairness. For users with higher channel gains over Nakagami-*m* fading channels, NOMA can offer higher individual rates than OMA. This was determined by an evaluation of the outage performance of NOMA with fixed power allocation in a downlink NOMA system, which was carried out by [17]. Most of the research that is currently available on cooperative NOMA was conducted under *p*-CSI conditions over Rayleigh fading channels. However, it is hard to use in real wireless systems due to mistakes in channel estimate. Furthermore, the Nakagami-*m* fading channel is employed. It has better statistical results than Rayleigh fading channels in several types of fading environments. The outage performance of cooperative NOMA with user relaying was investigated in [18] under Rayleigh fading. However, the impact of channel estimation errors on the performance of the system when operating across Nakagami-*m* fading channels was not acknowledged.

The authors of this study demonstrate that deploying RIS on the NOMA network can improve energy efficiency in NOMA [19]. As a result, the BS’s performance as a relay *decode and forward* (DF) could improve system reliability if adjacent users (those with better channel circumstances) perform well [20]. Because it offers a wide range of analysis, the authors employ the Nakagami-*m* fading model, where m is the fading parameter in [21]. Despite this, the Nakagami-*m* models will have a hard time representing transmission circumstances including LoS. The Nakagami-*m* model also has the advantage of being more analytical.

In this research, we take into consideration a downlink cooperative NOMA-RIS network with *p*-CSI that operates across Nakagami-*m* fading channels. Both of the following cooperative NOMA transmission possibilities are being discussed: in the first scenario, the base operation (BO) uses RIS to send information to users who are near and distant. In the second scenario, the BO can send information to both near and distant users using RIS, and, also, the distant user can receive data from a nearby user.

To look into how channel estimation errors affect wireless communication systems in use [22], we propose a downlink cooperative NOMA network with RIS. We evaluate NOMA users’ outage performance in two typical cooperative NOMA situations with respect to outage probability and over Nakagami-*m* fading channels.

We derive closed-form formulations of outage probability for a pair of NOMA users in two different scenarios, both of which include the user’s relaying information without a direct link. We have come up with rough expressions of the probability of an outage for a pair of NOMA users working in conditions with a high signal-to-noise ratio (SNR). This will help us learn more about how the network handles outages [4].

Closed-form equations of outage probability are derived for the RIS-aided NOMA system. The impact of each system parameter on the outage probability can be mathematically assessed because it is expressed in terms of numerous system parameters. The impact of the number of meta-surfaces in RIS, for example, on the possibility of outages, could be evaluated to understand how the system can improve its performance. This work indicates that the number of meta-surfaces in RIS determines the system’s outage probability. As a result, we derive the ergodic capacity for outage probability in both arbitrary and optimal phase shifts using references from [23,24,25,26,27,28]. The performance of a cooperative NOMA system over Nakagami-*m* fading channels under the conjecture of imperfect channel state information (*ip*-CSI) is explored in [21]. The closed-form expressions of outage probability for a pair of users are carefully generated to evaluate the outage performance of the two scenarios described. The purpose of this article [29] is to look at a new cooperative NOMA protocol in which the advanced relay lacks a fixed power source and works as a wirelessly powered relay to assist signal transmission to a representative weak user and strong user in NOMA. The principles of a 6G system are defined in [30], which presents a comprehensive, forward-looking view. Researchers believe that 6G will be more than just increased spectrum in high-frequency bands; rather, it will represent a convergence of forthcoming technological trends driven by innovative, underlying service concepts. The authors of [31] evaluate and compare the precise amount of powers required by various multiple-access strategies, demonstrating that NOMA does not fully conform to the stigmatizing superiority of NOMA over OMA in typical systems, such as orthogonal frequency division multiplexing (OFDM), a low-complexity approach for removing inter-symbol interference during transmission via frequency-selective fading channels [32,33].

## 3. System Model

**Figure 1:**
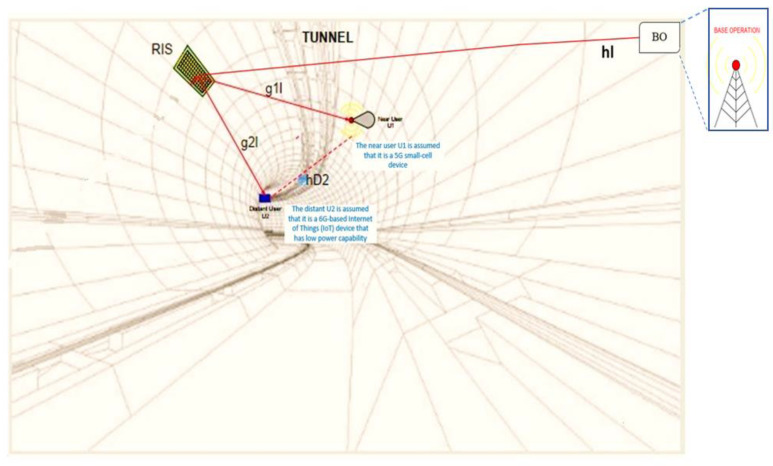
The system model. Notes: 1. The nearby user *U*_1_ is assumed to be a 5G small-cell device. 2. The distant *U*_2_ is assumed to be a 6G-based Internet of Things (IoT) device that has low power capability.

As illustrated in Figure 1, we examine a two-users method to NOMA downlink based on RIS. Orthogonal access is used to segregate numerous different groups of users. We assume there is a representative user, either nearby user *U*_1_ or distant user *U*_2_, in each category, where each user is categorized according to his location and power consumption ability. Furthermore, the distant user *U*_2_ is assumed to be a 6G-based Internet of Things (IoT) device that has low power capability, whereas the nearby user *U*_1_ is assumed to be a 5G small cell in this paper.

Due to significant blockage or obstruction, it was not possible to transmit directly from the base operation (BO) to users under certain circumstances. To serve two NOMA users, the BO generates two beamforming vectors using the zero-force beamforming technique. RIS-NOMA supports varied QoS needs by grouping paired users, making it useful for establishing multiple services.

With the help of an RIS that has *N* reflective elements, a controller for the substituting process, including operating modes, is also included in the RIS. For channel estimation, RIS works in the receiving mode, while, for data transmission, it works in the reflecting mode, due to the fact that the RIS is a passive reflecting system. On the device, a time-division duplexing (TDD) protocol is employed. For both uplink and downlink broadcasts, assume that the training procedure for the uplink will be used to collect channel information in the downlink. 

It is a new type of RIS implementation used on the NOMA network in this system that does not require a direct link from the BO to each user or a direct link from a nearby user to a distant user. Each link also considers errors induced by distance, which is novel in this study. The received signals from BO to *U*_1_ and *U*_2_ are denoted by $y_{{S,U}_{1}}$ and $y_{{S,U}_{2}}$, respectively. Those received signals $y_{{S,U}_{1}}$, $y_{{S,U}_{2}}$ are determined by equations provided in this section. The overlaid signal $y(t)=\sqrt{a_{1}P_{s}}x_{1}(t)+\sqrt{a_{2}P_{s}}x_{2}(t)$ transmitted from the BO is then required to supply distant mobile users with the presence of RIS in order to allow NOMA mode, where some key notations used in this paper are listed in Table 1. The NOMA concept is used in this study to offer user fairness, with *a*_1_ and *a*_2_ being the power allocation factors for users *U*_1_ and *U*_2_, respectively. We have a1 and *a*_1_ < *a*_2_ due to the lower amount of power required to feed user *U*_1_. All wireless links are considered to be suffering fading, attenuation caused by path loss distance, and exposed to additive white Gaussian noise (AWGN) with zero mean and *N*_0_ variance. We also assume that all links over Nakagami-*m* in wireless networks with perfect channel statistic information (*p*-CSI) have channel estimate errors.

## 4. Channel Distribution

We observe the behavior of the signals received by *U*_1_ and *U*_2_ by deriving the closed-form expression of each signal and simulating them. We use Nakagami-*m* as an approach to derive the closed-form expression.

By using this approach, we assume that each channel has an *identical independent distribution* (*i.i.d*). Next, each channel has a fading channel, which suffers attenuation by path-loss distance and noise. The links are arranged by channels. According to Figure 1, we define a link as consisting of the transmitted signal by BO to *U*_1_ through RIS. This link has two channels: one from the BO to the RIS and another from the RIS to *U*_1_.

Thus, the signal *y*(*t*) is transmitted from BO suffers from two fading channels, denoted by both $h_{l}$ and $g_{1l}$. The *h*_l_ is the fading coefficient of the channel of BO to RIS and $g_{1l}$ is the fading channel of RIS to *U*_1_. Furthermore, there are also other links consisting of the BO sending a signal to *U*_2_ through RIS so the transmitted signal from BO also suffers from two fading channels, i.e., $h_{l}$ and $g_{2l}$**.**

By using RIS, physically, the fading channel $h_{l}$ and $g_{1l}$ are related by $h_{l}^{H}\Phi g_{1l}$ relation, where Φ is denoted as phase shift. Based on the explanation above, $y_{{S,U}_{1}}$ is defined as the received signal by the *U*_1_, which also suffers the attenuation caused by path-loss distance and noise $n_{U_{1}}$ caused by propagation environment. Therefore, this phenomenon is formulated in Equation (1).

(1) $$y_{S,U_{1}}=\left[h_{l}^{H}\Phi g_{1l}\right]\left(\sqrt{a_{1}P_{s}}x_{1}+\sqrt{a_{2}P_{s}}x_{2}\right)+n_{U_{1}}$$

Because RIS is made up of multiple elements (e.g., *N* elements), each of which receives and reflects signals, the term $h_{l}^{H}\Phi g_{1l}$ could be expressed as Equation (2) by assuming the reflection amplitude coefficient α is considered to be 1, which indicates lossless reflection. The symbol $\theta_{n}$ in Equation (2) represents the adjustable phase that is used on each *N*^th^ element of RIS. In practice, every channel suffers the Nakagami–m fading, which probably has error; then, each fading channel could be represented by $h_{l}={\hat{h}}_{l}+e_{l}$ and $g_{vl}={\hat{g}}_{vl}+e_{vl}$. The $h_{l}$ and $g_{vl}$ are fading channels of link *v*, and ${\hat{h}}_{l}$, ${\hat{g}}_{vl}$ are the average fading channels of link *v*.

Apart from this, it also suffers an additive white Gaussian noise (AWGN) with zero mean and variance *N*_0_. Based on the aforementioned, by assuming that ${\hat{h}}_{l}$, ${\hat{g}}_{vl}$, $e_{l}$, and $e_{vl}$ are statistically independent, then we could state the ${\hat{h}}_{l}\sim \mathbb{C}\mathbb{N}\left(0,\beta_{SR}I_{N}\right)$, ${\hat{g}}_{vl}\sim \mathbb{C}\mathbb{N}\left(0,\beta_{Rv}I_{N}\right)$, where $\theta_{n}\in \left[-\pi ,\pi \right]$, $I_{N}$, and $\phi =diag\left(e^{j\theta_{1}},e^{j\theta_{2}},\ldots ,e^{j\theta_{N}}\right)$ are identity matrices with order *N* with range of $\theta_{n}$, respectively. In addition, error could be represented as $e_{l}\sim \mathbb{C}\mathbb{N}\left(0,\sigma_{e_{l}}^{2}I_{N}\right)$, $e_{vl}\sim \mathbb{C}\mathbb{N}\left(0,\sigma_{e_{vl}}^{2}I_{N}\right)$, and $n_{v}\sim \mathbb{C}\mathbb{N}\left(0,N_{0}I_{N}\right)$.

Average power of channel is defined as $E\left\{{\left|{\hat{h}}_{l}\right|}^{2}\right\}=\Omega_{l}$ and $E\left\{{\left|{\hat{g}}_{vl}\right|}^{2}\right\}=\Omega_{vl}$, where $\Omega_{l}={\hat{\Omega }}_{l}+e_{l}$ and $\Omega_{vl}={\hat{\Omega }}_{vl}+e_{vl}$, respectively. If we also define $\eta_{SR}=\sigma_{e_{l}}^{2}/\Omega_{l}$ and $\eta_{RU_{v}}=\sigma_{e_{vl}}^{2}/\Omega_{vl}$, then average power of channel ${\hat{\Omega }}_{l}=\left(1-\eta_{SR}\right)\;d_{SR}^{-\chi }$ and ${\hat{\Omega }}_{vl}=\left(1-\eta_{RU_{v}}\right)\;d_{RU_{v}}^{-\chi }$. By considering this reason, Equation (2) could be written as Equation (3).

(2) $$y_{S,U_{1}}=\left[\alpha \sum_{l=1}^{N}h_{l}g_{1l}e^{j\theta_{l}}\right]\left(\sqrt{a_{1}P_{s}}x_{1}+\sqrt{a_{2}P_{s}}x_{2}\right)+n_{U_{1}}$$

(3) $$y_{S,U_{1}}=\left(\sum_{l=1}^{N}\left|{\hat{h}}_{l}+e_{1}\right|\left|{\hat{g}}_{1l}+e_{1}\right|\right)\;\left(\sqrt{a_{1}P_{s}}x_{1}+\sqrt{a_{2}P_{s}}x_{2}\right)+n_{U_{1}}$$

Similarly, the distant user *U*_2_ receives signals through RIS ($BS\to RIS\to U_{2}$),denoted as $y_{S,U_{2}}$ and shown in Equation (4).

(4) $$y_{S,U2}=\left[\left(\sum_{l=1}^{N}\left|{\hat{h}}_{l}+e_{2l}\right|\left|{\hat{g}}_{2l}+e_{2l}\right|\right)\left(\sqrt{a_{1}P_{s}}x_{1}+\sqrt{a_{2}P_{s}}x_{2}\right)\right]+n_{U_{2}}$$

### 4.1. Near User

From the NOMA concept, the *x*_2_ signal is decoded using sequential interference cancellation (SIC) of the received superposition signal at *x*_1_, where the higher transmit power of *x*_2_ results in less inter-user interference. The interference and noise ratio (SINR) for *U*_1_ could be expressed in Equation (5).

(5) $$\rho {(}_{U_{1,}x_{2}\to x_{1}})=\frac{\left({\left|\sum_{l=1}^{N}\left|{\hat{h}}_{l}\right|\left|{\hat{g}}_{1l}\right|\right|}^{2}\right)a_{2}P_{s}}{\left({\left|\sum_{l=1}^{N}\left|{\hat{h}}_{l}\right|\left|{\hat{g}}_{1l}\right|\right|}^{2}a_{1}+\left(\frac{\sigma_{l}^{2}}{N}+\frac{\sigma_{1l}^{2}}{N}\right)\right)P_{s}+N_{0}}$$

By assuming the term $\left(\frac{\sigma_{l}^{2}}{N}+\frac{\sigma_{1l}^{2}}{N}\right)P_{s}$ in Equation (5) is errors due to path-loss distance, then that term could be mentioned as $\frac{\eta_{SR_{1}}}{N}\left(\;d_{SR_{1}}^{-\chi }\right).\frac{\eta_{RU_{1}}}{N}\left(\;d_{RU_{1}}^{-\chi }\right)P_{s}$. Therefore, Equation (5) could be expressed as Equation (6).

(6) $$\rho {(}_{U_{1,}x_{2}\to x_{1}})=\frac{\left({\left|\sum_{l=1}^{N}\left|{\hat{h}}_{l}\right|\left|{\hat{g}}_{1l}\right|\right|}^{2}\right)a_{2}\rho_{s}}{\left({\left|\sum_{l=1}^{N}\left|{\hat{h}}_{l}\right|\left|{\hat{g}}_{1l}\right|\right|}^{2}\right)a_{1}\rho_{s}+\frac{\eta_{SR}}{N}\left(\;d_{SR}^{-\chi }\right).\frac{\eta_{RU_{1}}}{N}\left(\;d_{RU_{1}}^{-\chi }\right)\rho_{s}+1}$$

Meanwhile, Equation (7) gives the received SINR for *U*_1_ to decode its own signal: *x*_1_.

(7) $$\rho {(}_{U_{1,}x_{1}})=\frac{\left({\left|\sum_{l=1}^{N}\left|{\hat{h}}_{l}\right|\left|{\hat{g}}_{1l}\right|\right|}^{2}\right)a_{1}P_{s}}{\left({\left|\sum_{l=1}^{N}\left|{\hat{h}}_{l}\right|\left|{\hat{g}}_{1l}\right|\right|}^{2}a_{1}+\left(\frac{\sigma_{l}^{2}}{N}+\frac{\sigma_{1l}^{2}}{N}\right)\right)P_{s}+N_{0}}$$

Next, it could be expressed in Equation (8).

(8) $$\rho {(}_{U_{1,}x_{1}})=\frac{\left({\left|\sum_{l=1}^{N}\left|{\hat{h}}_{l}\right|\left|{\hat{g}}_{1l}\right|\right|}^{2}\right)a_{1}\rho_{s}}{\left({\left|\sum_{l=1}^{N}\left|{\hat{h}}_{l}\right|\left|{\hat{g}}_{1l}\right|\right|}^{2}\right)a_{1}\rho_{s}+\frac{\eta_{SR}}{N}\left(\;d_{SR}^{-\chi }\right).\frac{\eta_{RU_{1}}}{N}\left(\;d_{RU_{1}}^{-\chi }\right)\rho_{s}+1}$$

Assuming that $\zeta_{1}$ and $\zeta_{2}$ represent the appropriate target levels for *U*_1_ and *U*_2_, we can state the two SNR thresholds $\rho_{Th_{1}}$ and $\rho_{Th_{2}}$ as Equations (9) and (10), respectively.

(9) $$\rho_{Th_{1}}=\frac{\left(\;2^{2\zeta_{1}}-1\right)}{\rho_{s}}$$

(10) $$\rho_{Th_{2}}=\frac{\left(\;2^{2\zeta_{2}}-1\right)}{\rho_{s}}$$

The $\tau_{1}$ is defined as *first*-*comparison*-*parameter* against the SNR threshold, which could be derived from Equation (8) so that Equation (11) is obtained. Similarly, we could express $\tau_{2}$ as *second*-*comparison*-*parameter* that could be derived from Equation (6) and obtained as Equation (12). (*Proof.* Please see Appendix A.)

(11) $$\tau_{1}=\frac{\rho_{Th_{1}}\lambda_{1}}{\left(a_{1}-a_{1}\rho_{Th_{1}}\right)\rho_{s}}$$

(12) $$\tau_{2}=\frac{\rho_{Th_{2}}\lambda_{1}}{\left(a_{2}-a_{1}\rho_{Th_{2}}\right)\rho_{s}}$$

*Outage Probability*: a parameter $X_{v}$ is defined as a gain of the channel coefficient of link *v* caused by RIS implementation. If the elements of RIS have arbitrary phase shifts condition, then it is defined as $X_{v}=|h_{l}^{H}\phi g_{vl}|$.

Furthermore, we determine that $E\{X_{v}^{2}\}=E\{|Xv+e{|}^{2}\}$, expressed by Equation (13), and variance $\mathrm{Var}(X_{v})$ expressed by Equation (14) by defining $\beta_{SR_{v}}$ and $\beta_{RU_{v}}$ are the large scale of fading channel $SU_{v}$, $SR_{v}$, and $RU_{v}$ respectively.

(13) $$E\left\{{\left|X_{v}\right|}^{2}\right\}=(2N^{2}+2N)\beta_{RU_{v}}^{2}\beta_{SR}^{2}+{\left|\frac{\eta_{SR}}{N}\left(\;d_{SR}^{-\chi }\right).\frac{\eta_{RU_{v}}}{N}\left(\;d_{RU_{v}}^{-\chi }\right)\right|}^{2}$$

(14) $$Var\left\{\left|X_{v}\right|\right\}=\left(N^{2}+2N\right)\beta_{SR}^{2}\beta_{RU_{v}}^{2}-2N\beta_{SR}\beta_{RU_{v}}\left|\frac{\eta_{SR}}{N}\left(\;d_{SR_{2}}^{-\chi }\right).\frac{\eta_{RU_{v}}}{N}\left(\;d_{RU_{v}}^{-\chi }\right)\right|$$

Equation (15) is used to represent *m_v_* as the shape factor of the large-scale fading channel on link *v*:

(15) $$m_{\upsilon }=\frac{E\left\{{\left|X_{\upsilon }\right|}^{2}\right\}}{Var\left(X_{\upsilon }\right)}$$

where index of *v* shows link *v* ∈{1,2}, so that *m*_1_ and *m*_2_ are the shape factor of the large scale of fading channel on *U*_1_ and *U*_2_, respectively.

By assuming that parameter *m_v_* ≥ 1, the PDF and CDF could be written as Equations (16) and (17).

(16) $$f_{{\left|{\hat{X}}_{v}\right|}^{2}}\left(\tau \right)={\left(\delta_{v}\right)}^{m_{v}}\frac{\tau^{m_{v}-1}}{\left(m_{v}-1\right)!}e^{-\delta_{v}\tau }$$

(17) $$F_{{\left|{\hat{X}}_{v}\right|}^{2}}\left(\tau \right)=1-e^{-\delta_{v}\tau }\sum_{i-0}^{m_{v}-1}\frac{1}{i!}{\left(\delta_{v}\tau \right)}^{i}$$

In this study, the events that complement the outage occur at *U*_1_. When *U*_1_ successfully decodes both the signal *x*_2_ and its own signal *x*_1_, the outage probability of *U*_1_ could be expressed as Equation (18):

(18) $$P_{U_{1}}=1-\mathrm{Pr}\left(\rho {(}_{U_{1,}x_{2}\to x_{1}})>\rho_{Th_{2}},\rho {(}_{U_{1,}x_{1}})>\rho_{Th_{1}}\right)$$

Because each channel is suffering attenuation caused by path-loss distance and noise, we then define a parameter $\lambda_{1}$ wherein this parameter can determine both values of $\tau_{1}$ and $\tau_{2}$, respectively, in Equation (11), Equation (12). The value of $\lambda_{1}$ is determined by using Equation (19).

(19) $$\lambda_{1}=\frac{\eta_{SR}}{N}\left(\;d_{SR}^{-\chi }\right).\frac{\eta_{RU_{1}}}{N}\left(\;d_{RU_{1}}^{-\chi }\right)\rho_{s}+1$$

By defining *a*_1_ + *a*_2_ = 1 where *a*_2_ > *a*_1_, then Equation (18) could be expressed as Equation (20),

(20) $$P_{U_{1}}=1-e^{-\delta_{1}\tau }\sum_{j-0}^{m_{1}-1}\frac{{\left(\delta_{1}\tau \right)}^{j}}{j!}$$

where the values of *m*_1_, and *δ*_1_ are found by using Equation (21), and Equation (22), respectively,

(21) $$m_{1}=\frac{(2N^{2}+2N)\beta_{RU_{1}}^{2}\beta_{SR}^{2}+{\left|\frac{\eta_{SR}}{N}\left(\;d_{SR}^{-\chi }\right).\frac{\eta_{RU_{v}}}{N}\left(\;d_{RU_{1}}^{-\chi }\right)\right|}^{2}}{(N^{2}+2N)\beta_{RU_{1}}^{2}\beta_{SR}^{2}-2N\beta_{SR}\beta_{RU_{1}}\frac{\eta_{SR}}{N}\left(\;d_{SR}^{-\chi }\right).\frac{\eta_{RU_{v}}}{N}\left(\;d_{RU_{1}}^{-\chi }\right)}$$

(22) $$\delta_{1}=\frac{m_{1}}{N}\left(\frac{\;d_{SR}^{\chi }}{1-\eta_{SR}}+\frac{\;d_{RU_{1}}^{\chi }}{1-\eta_{RU_{1}}}\right)$$

while τ is determined by $\tau =\max(\tau_{2},\tau_{1})$.

Next, the outage probability at *U*_1_ could be rewritten in Equation (23).

(23) $$P_{U_{1}}=1-e^{-\frac{m_{1}}{N}\left(\frac{\;d_{SR}^{\chi }}{1-\eta_{SR}}+\frac{\;d_{RU_{1}}^{\chi }}{1-\eta_{RU_{1}}}\right)\tau }\sum_{j-0}^{m_{1}-1}\frac{{\left(\frac{m_{1}}{N}\left(\frac{\;d_{SR}^{\chi }}{1-\eta_{SR}}+\frac{\;d_{RU_{1}}^{\chi }}{1-\eta_{RU_{1}}}\right)\tau \right)}^{j}}{j!}$$

*Proof.* Please see Appendix A.

### 4.2. Distant User

To derive the mathematical expression of the outage probability at *U*_2_, several steps must be completed. First, *U*_2_ only needs to deal with the signal *x*_1_ of *U*_1_ as noise and decode its own signal *x*_2_, which is expressed in Equation (7). Meanwhile, in the second condition, the received signal of *U*_2_ from BS through RIS could be expressed in Equation (6). According to the system model which is depicted in Figure 1, *U*_2_ receives signals from two different links. The first slot is a link from BS to *U*_2_ through RIS and a relaying link from *U*_1_ in the second slot. The total received signal at *U*_2_ is expressed as Equation (25), which denotes the received SINR after selection combining (SC) at *U*_2_, i.e., summing of $\rho_{U_{2,}x_{2}}$ in Equation (24) and $\rho_{U_{1,}x_{2}\to x_{1}}$ in Equation (6).

(24) $$\rho_{U_{2,}x_{2}}=\frac{\left({\left|\sum_{l=1}^{N}\left|{\hat{h}}_{l}\right|\left|{\hat{g}}_{2l}\right|\right|}^{2}\right)a_{2}\rho_{s}}{\left({\left|\sum_{l=1}^{N}\left|{\hat{h}}_{l}\right|\left|{\hat{g}}_{2l}\right|\right|}^{2}\right)a_{1}\rho_{s}+\frac{\eta_{SR}}{N}\left(\;d_{SR}^{-\chi }\right).\frac{\eta_{RU_{2}}}{N}\left(\;d_{RU_{2}}^{-\chi }\right)\rho_{s}+1}$$

(25) $$\rho_{U_{2}}^{SC}=\frac{\left({\left|\sum_{l=1}^{N}\left|{\hat{h}}_{l}\right|\left|{\hat{g}}_{1l}\right|\right|}^{2}\right)a_{2}\rho_{s}}{\left({\left|\sum_{l=1}^{N}\left|{\hat{h}}_{l}\right|\left|{\hat{g}}_{1l}\right|\right|}^{2}\right)a_{1}\rho_{s}+\frac{\eta_{SR}}{N}\left(\;d_{SR}^{-\chi }\right).\frac{\eta_{RU_{1}}}{N}\left(\;d_{RU_{1}}^{-\chi }\right)\rho_{s}+1}+\frac{\left({\left|\sum_{l=1}^{N}\left|{\hat{h}}_{l}\right|\left|{\hat{g}}_{2l}\right|\right|}^{2}\right)a_{2}\rho_{s}}{\left({\left|\sum_{l=1}^{N}\left|{\hat{h}}_{l}\right|\left|{\hat{g}}_{2l}\right|\right|}^{2}\right)a_{1}\rho_{s}+\frac{\eta_{SR}}{N}\left(\;d_{SR}^{-\chi }\right).\frac{\eta_{RU_{2}}}{N}\left(\;d_{RU_{2}}^{-\chi }\right)\rho_{s}+1}$$

*Outage Probability*: the *U*_2_ outage events occur if one of the two occurrences below happens. When *U*_1_ is unable to decode the signal *x*_2_, the first event happens. The second event occurs when *U*_2_ could not decode its own signal *x*_2_ despite *U*_1_ being able to decode the signal *x*_2_. From the above occurrences, the outage probability of *U*_2_ might be stated by Equations (26)–(28).

(26) $$P_{U_{2}}=\mathrm{Pr}\left(\rho {(}_{U_{1,}x_{2}\to x_{1}})<\rho_{Th_{2}}\right)+\mathrm{Pr}\left(\rho_{2,U_{2}}<\rho_{Th_{2}},\rho {(}_{U_{1,}x_{2}\to x_{1}})>\rho_{Th_{2}}\right)$$

(27) $$P_{U_{2}}=1-e^{\left(\delta_{1}\tau_{2}-\delta_{2}\tau_{3}\right)}\sum_{j-0}^{m_{1}-1}\sum_{k-0}^{m_{2}-1}\frac{{\left(\delta_{1}\tau_{2}\right)}^{j}{\left(\delta_{2}\tau_{3}\right)}^{k}}{j!k!},\mathrm{where}$$

(28) $$m_{2}=\frac{(2N^{2}+2N)\beta_{RU_{2}}^{2}\beta_{SR}^{2}+{\left|\frac{\eta_{SR}}{N}\left(\;d_{SR}^{-\chi }\right).\frac{\eta_{RU_{v}}}{N}\left(\;d_{RU_{2}}^{-\chi }\right)\right|}^{2}}{(N^{2}+2N)\beta_{RU_{2}}^{2}\beta_{SR}^{2}-2N\beta_{SR}\beta_{RU_{2}}\frac{\eta_{SR}}{N}\left(\;d_{SR}^{-\chi }\right).\frac{\eta_{RU_{v}}}{N}\left(\;d_{RU_{2}}^{-\chi }\right)}$$

Similarly, $\tau_{3}$ is defined as *third*-*comparison*-*parameter* against SNR threshold of *U*_2_, which could be derived from Equation (29). In addition, attenuation and noise $\lambda_{2}$ suffered by received signal by *U*_2_ could be expressed in Equation (30).

(29) $$\tau_{3}=\frac{\rho_{Th_{2}}\lambda_{2}}{\left(a_{2}-a_{1}\rho_{Th_{2}}\right)\rho_{s}}$$

(30) $$\lambda_{2}=\frac{\eta_{SR}}{N}\left(\;d_{SR}^{-\chi }\right).\frac{\eta_{RU_{2}}}{N}\left(\;d_{RU_{2}}^{-\chi }\right)\rho_{s}+1$$

It is also defined that the scale factor of the gamma distribution of the channel $\delta_{2}$ at *U*_2_ could be expressed in Equation (31).

(31) $$\delta_{2}=\frac{m_{2}}{N}\left(\left(\frac{\;d_{SR}^{\chi }}{\left(1-\eta_{SR}\right)}\times \frac{\;d_{RU_{2}}^{\chi }}{\left(1-\eta_{RU_{2}}\right)}\right)\right)$$

*Proof*. Please see Appendix B.

## 5. Ergodic Capacity

The ergodic capacity is independent of small-scale fading on a long timescale. By implementing RIS in the NOMA network, we could formulate the ergodic capacity at *U*_1_ and *U*_2_. The general form of the instantaneous channel capacity (b/s/Hz), by assuming coherent combination, is stated in Equation (32).

(32) $$C=\left\{\log_{2}\left(1+\rho \right)\right\}$$

There are two conditions of phase shifts which are evaluated, i.e., arbitrary and the optimal phase shifts. For the arbitrary phase shifts, the instantaneous channel capacity at *U*_1_ is expressed in Equation (33).

(33) $$C_{U_{1}}{}_{Abtr}=\log_{2}\left(1+\frac{\left({\left|{\hat{h}}_{l}^{H}\Phi {\hat{g}}_{1l}\right|}^{2}\right)a_{2}\rho_{s}}{\left({\left|{\hat{h}}_{l}^{H}\Phi {\hat{g}}_{1l}\right|}^{2}\right)a_{1}\rho_{s}+\lambda_{1}}\right)$$

In addition, the instantaneous channel capacity at *U*_2_ is expressed in Equation (34). Furthermore, for the optimal phase shifts, the instantaneous channel capacity at *U*_1_ is expressed in Equation (35) and the instantaneous channel capacity at *U*_2_ in Equation (36).

(34) $$C_{U_{2}}{}_{Abtr}=\log_{2}\left(1+\frac{\left({\left|{\hat{h}}_{l}^{H}\Phi {\hat{g}}_{1l}\right|}^{2}\right)a_{2}\rho_{s}}{\left({\left|{\hat{h}}_{l}^{H}\Phi {\hat{g}}_{1l}\right|}^{2}\right)a_{1}\rho_{s}+\lambda_{1}}+\frac{\left({\left|{\hat{h}}_{l}^{H}\Phi {\hat{g}}_{2l}\right|}^{2}\right)a_{2}\rho_{s}}{\left({\left|{\hat{h}}_{l}^{H}\Phi {\hat{g}}_{2l}\right|}^{2}\right)a_{1}\rho_{s}+\lambda_{2}}\right)$$

(35) $$C_{U_{1}}{}_{Op}=\log_{2}\left(1+\frac{\left({\left|\sum_{l=1}^{N}\left|{\hat{h}}_{l}\right|\left|{\hat{g}}_{1l}\right|\right|}^{2}\right)a_{2}\rho_{s}}{\left({\left|\sum_{l=1}^{N}\left|{\hat{h}}_{1l}\right|\left|{\hat{g}}_{1l}\right|\right|}^{2}\right)a_{1}\rho_{s}+\lambda_{1}}\right)$$

(36) $$C_{U_{2}}{}_{Op}=\log_{2}\left(1+\frac{\left({\left|\sum_{l=1}^{N}\left|{\hat{h}}_{l}\right|\left|{\hat{g}}_{1l}\right|\right|}^{2}\right)a_{2}\rho_{s}}{\left({\left|\sum_{l=1}^{N}\left|{\hat{h}}_{l}\right|\left|{\hat{g}}_{1l}\right|\right|}^{2}\right)a_{1}\rho_{s}+\lambda_{1}}+\frac{\left({\left|\sum_{l=1}^{N}\left|{\hat{h}}_{l}\right|\left|{\hat{g}}_{2l}\right|\right|}^{2}\right)a_{2}\rho_{s}}{\left({\left|\sum_{l=1}^{N}\left|{\hat{h}}_{l}\right|\left|{\hat{g}}_{2l}\right|\right|}^{2}\right)a_{1}\rho_{s}+\lambda_{2}}\right)$$

Averaging over several different instantaneous channel realizations is one native technique to evaluating the ergodic capabilities in Equations (33)–(36), but it needs significant computational cost and is especially difficult with a large RIS array. We computed the ergodic capacity in closed-form to get around the problem, so that it could derive the ergodic capacity of *U*_1_ and *U*_2_.

### 5.1. Ergodic Capacity Evaluation for U_1_

According to Equation (33), we define the ergodic capacity of *U*_1_ for arbitrary phase shifts, which could be expressed in Equations (37) and (38).

(37) $${\hat{C}}_{U_{1}Abtr}=E\left\{\log_{2}\left(1+\frac{\left({\left|{\hat{h}}_{l}^{H}\Phi {\hat{g}}_{1l}\right|}^{2}\right)a_{2}\rho_{s}}{\left({\left|{\hat{h}}_{l}^{H}\Phi {\hat{g}}_{1l}\right|}^{2}\right)a_{1}\rho_{s}+\lambda_{1}}\right)\right\}$$

Furthermore, from Equation (35), the ergodic capacity for optimal phase shifts could be mentioned in Equations (39) and (40).

(38) $${\hat{C}}_{U_{1}Abtr}=\log_{2}\left(1+\frac{a_{2}\rho_{s}(2N^{2}+2N)\beta_{RU}^{2}\beta_{SR}^{2}}{a_{1}\rho_{s}(2N^{2}+2N)\beta_{RU_{1}}^{2}\beta_{SR}^{2}+\left(\frac{\eta_{SR}}{N}\left(\;d_{SR}^{-\chi }\right).\frac{\eta_{RU_{1}}}{N}\left(\;d_{RU_{1}}^{-\chi }\right)\right)\rho_{s}+1}\right)$$

(39) $${\hat{C}}_{U_{1}Op}=E\left\{\log_{2}\left(1+\frac{\left({\left|\sum_{l=1}^{N}\left|{\hat{h}}_{l}\right|\left|{\hat{g}}_{1l}\right|\right|}^{2}\right)a_{2}\rho_{s}}{\left({\left|\sum_{l=1}^{N}\left|{\hat{h}}_{1l}\right|\left|{\hat{g}}_{1l}\right|\right|}^{2}\right)a_{1}\rho_{s}+\lambda_{1}}\right)\right\}$$

(40) $${\hat{C}}_{U_{1}Op}=\log_{2}\left(1+\frac{\left(\left(N\left(1-\frac{\pi^{2}}{16}\right)\right)\beta_{RU_{2}}\beta_{SR}+\frac{N^{2}\pi^{2}}{16}\beta_{RU_{2}}\beta_{SR}\right)a_{2}\rho_{s}}{\left(\left(N\left(1-\frac{\pi^{2}}{16}\right)\right)\beta_{RU_{1}}\beta_{SR}+\frac{N^{2}\pi^{2}}{16}\beta_{RU_{1}}\beta_{SR}\right)a_{1}\rho_{s}+\left(\frac{\eta_{SR}}{N}\left(\;d_{SR}^{-\chi }\right).\frac{\eta_{RU_{2}}}{N}\left(\;d_{RU_{1}}^{-\chi }\right)\right)\rho_{s}+1}\right)$$

*Proof.* Please see Appendix C.

### 5.2. Ergodic Capacity Evaluation for U_2_

Similarly, from Equation (34), the ergodic capacity of *U*_2_ for arbitrary phase shifts is expressed in Equation (41). By defining $|{\hat{h}}_{l}^{H}\Phi {\hat{g}}_{1l}{|}^{2}$ as $(2N^{2}+2N)\beta_{RU_{1}}^{2}\beta_{SR}^{2}$ and $|{\hat{h}}_{l}^{H}\Phi {\hat{g}}_{2l}{|}^{2}$ as $(2N^{2}+2N)\beta_{RU_{2}}^{2}\beta_{SR}^{2}$, then Equation (41) could be rewritten as Equation (42).

(41) $${\hat{C}}_{U_{2}Abtr}=\log_{2}\left(1+E\left\{\frac{\left({\left|{\hat{h}}_{l}^{H}\Phi {\hat{g}}_{1l}\right|}^{2}\right)a_{2}\rho_{s}}{\left({\left|{\hat{h}}_{l}^{H}\Phi {\hat{g}}_{1l}\right|}^{2}\right)a_{1}\rho_{s}+\lambda_{1}}+\frac{\left({\left|{\hat{h}}_{l}^{H}\Phi {\hat{g}}_{2l}\right|}^{2}\right)a_{2}\rho_{s}}{\left({\left|{\hat{h}}_{l}^{H}\Phi {\hat{g}}_{2l}\right|}^{2}\right)a_{1}\rho_{s}+\lambda_{2}}\right\}\right)$$

(42) $${\hat{C}}_{U_{2}Abtr}=\log_{2}\left(1+\frac{a_{2}\rho_{s}\left(2N^{2}+2N\right)\beta_{RU_{1}}^{2}\beta_{SR}^{2}}{a_{1}\rho_{s}\left(2N^{2}+2N\right)\beta_{RU_{1}}^{2}\beta_{SR}^{2}+\lambda_{1}}+\frac{a_{2}\rho_{s}\left(2N^{2}+2N\right)\beta_{RU_{2}}^{2}\beta_{SR}^{2}}{a_{1}\rho_{s}\left(2N^{2}+2N\right)\beta_{RU_{2}}^{2}\beta_{SR}^{2}+\lambda_{2}}\right)$$

Moreover, from Equation (36), the ergodic capacity of *U*_2_ for optimal phase shifts could be expressed in Equation (43). If substituting the same terms ${\left|{\hat{h}}_{l}^{H}\Phi {\hat{g}}_{1l}\right|}^{2}$ by $\left(N\left(1-\frac{\pi^{2}}{16}\right)\right)\beta_{RU_{1}}\beta_{SR}+\frac{N^{2}\pi^{2}}{16}\beta_{RU_{1}}\beta_{SR}$ and ${\left|{\hat{h}}_{l}^{H}\Phi {\hat{g}}_{2l}\right|}^{2}$ by $\left(N\left(1-\frac{\pi^{2}}{16}\right)\right)\beta_{RU_{2}}\beta_{SR}+\frac{N^{2}\pi^{2}}{16}\beta_{RU_{2}}\beta_{SR}$ in Equation (43), then Equation (44) could be obtained below.

(43) $${\hat{C}}_{U_{2}Op}=\log_{2}\left(1+E\left\{\frac{\left({\left|\sum_{l=1}^{N}\left|{\hat{h}}_{l}\right|\left|{\hat{g}}_{1l}\right|\right|}^{2}\right)a_{2}\rho_{s}}{\left({\left|\sum_{l=1}^{N}\left|{\hat{h}}_{l}\right|\left|{\hat{g}}_{1l}\right|\right|}^{2}\right)a_{1}\rho_{s}+\lambda_{1}}+\frac{\left({\left|\sum_{l=1}^{N}\left|{\hat{h}}_{l}\right|\left|{\hat{g}}_{2l}\right|\right|}^{2}\right)a_{2}\rho_{s}}{\left({\left|\sum_{l=1}^{N}\left|{\hat{h}}_{l}\right|\left|{\hat{g}}_{2l}\right|\right|}^{2}\right)a_{1}\rho_{s}+\lambda_{2}}\right\}\right)$$

(44) $${\hat{C}}_{U_{2}Op}=\log_{2}\left(\begin{array}{l} 1+\frac{\left(\left(N\left(1-\frac{\pi^{2}}{16}\right)\right)\beta_{RU_{1}}\beta_{SR}+\frac{N^{2}\pi^{2}}{16}\beta_{RU_{1}}\beta_{SR}\right)a_{2}\rho_{s}}{\left(\left(N\left(1-\frac{\pi^{2}}{16}\right)\right)\beta_{RU_{1}}\beta_{SR}+\frac{N^{2}\pi^{2}}{16}\beta_{RU_{1}}\beta_{SR}\right)a_{1}\rho_{s}+\lambda_{1}}\\ +\frac{\left(\left(N\left(1-\frac{\pi^{2}}{16}\right)\right)\beta_{RU_{2}}\beta_{SR}+\frac{N^{2}\pi^{2}}{16}\beta_{RU_{2}}\beta_{SR}\right)a_{2}\rho_{s}}{\left(\left(N\left(1-\frac{\pi^{2}}{16}\right)\right)\beta_{RU_{2}}\beta_{SR}+\frac{N^{2}\pi^{2}}{16}\beta_{RU_{2}}\beta_{SR}\right)a_{1}\rho_{s}+\lambda_{2}} \end{array}\right)$$

*Proof*. Please see Appendix D.

## 6. Result and Discussion

The outage performance of the RIS-aided NOMA network with *p*-CSI in terms of probable outages via the Nakagami-*m* fading channel is evaluated numerically in this section. Table 2 sets the simulation parameters, including in its numerical definition. Furthermore, MATLAB programing software is used to create simulations by adjusting parameters. The exact expressions of the outage probability are verified using Monte Carlo simulations.

**Figure 2:**
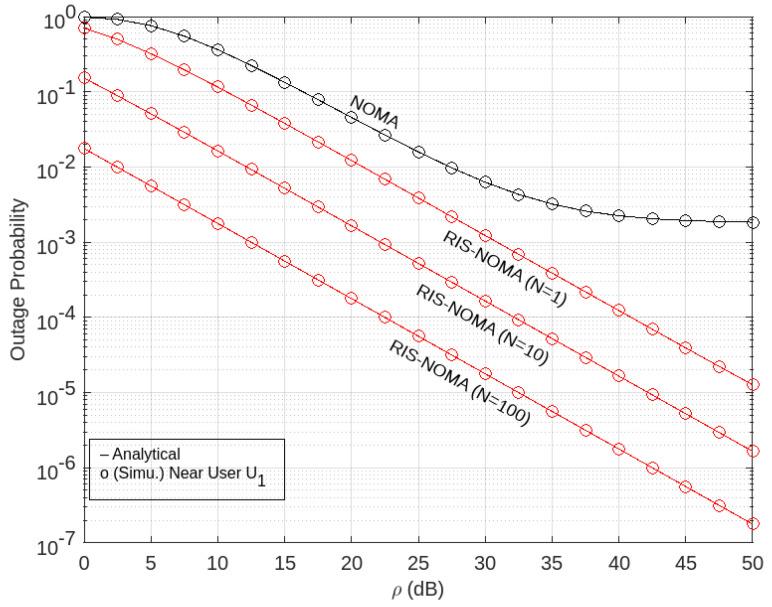
Outage probability versus transmit SNR at *U*_1_.

The results of the foregoing investigations were confirmed numerically using a system in which the locations were mapped into the Cartesian co-ordinate system. The source (BO) is at (0, 0), whereas the RIS is at (60, 10), assuming that the positions of BO, *U*_1_, and *U*_2_ in Figure 2 are a straight line.

**Figure 3:**
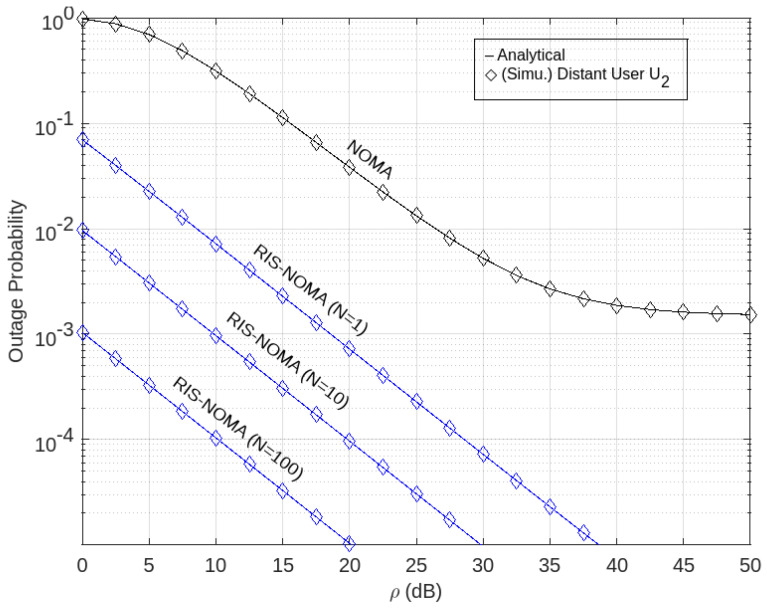
Outage probability versus transmit SNR at *U*_2_.

**Figure 4:**
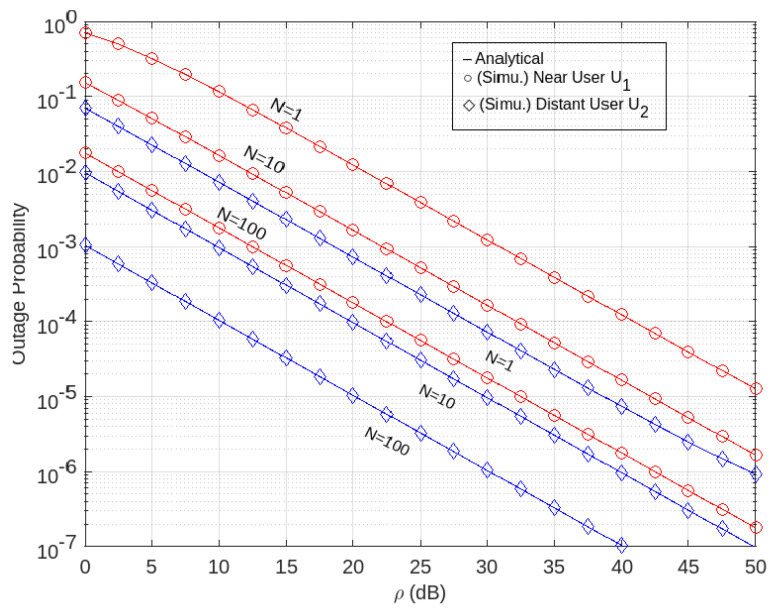
Comparison of outage probability versus transmit SNR between *U*_1_ and *U*_2_.

The distance between BO and *U*_2_ in this paper is normalized to one. The outage probability of users for the non-direct connection scenario from BS is plotted against the transmitted SNR in Figure 2, Figure 3 and Figure 4. The results of Monte Carlo simulation is also shown, which are given marks of circle and diamond, respectively.

For the users *U*_1_ and *U*_2_, we set the goal rates as ζ_1_ = 3.6 and ζ_2_ = 1 *BPCU* (*Bit Per Channel Use*), respectively. By entering the values of *m*_1_ and *m*_2_, according to Equations (21) and (28), then the exact outage probability curves of the pair of users are plotted based on Equation (23) for *U*_1_ and Equation (27) for *U*_2_.

In addition, Figure 2 shows the simulation results of the outage probability against the SNR by implementing RIS on the NOMA network for the number of different RIS elements for users near *U*_1_. The figure shows the difference in performance between the RIS-aided NOMA, as shown by the red graph, and the NOMA performance shown by the black graph. Here, it can be seen that the RIS-aided NOMA network has a lower probability of outage value than the NOMA network, which means that the RIS-aided NOMA network has better performance than the NOMA network for closest users, which has important meaning in networks that use NOMA-based concepts because it functions as a relay. Furthermore, from the figure, it can be seen that the greater the number of RIS elements, the smaller the outage probability. This means that the greater the number of RIS elements, the better the performance.

Moreover, Figure 3 shows the simulation and results of the outage probability analysis on the SNR for remote users of *U*_2_. In the figure, it can be seen that the higher the number of RIS elements, the lower the outage probability value, which means the more RIS elements, the better the performance. In addition, it is also seen that, when compared to the performance of the network system using NOMA, the implementation of RIS on the NOMA network outperforms it. This can be seen from the graph where the blue graph representing the performance of NOMA assisted by RIS has a lower outage probability value than the black graph representing the performance of NOMA.

Further, if we combine the profiles in Figure 2 and Figure 3, it is seen that the far-user outage probability, *U*_2_, outperforms the near-user outage probability, *U*_1_. This can be seen from Figure 4, which shows a comparison of the outage probability to the SNR for near users *U*_1_ and far users *U*_2_.

It can be seen that the probability of SNR blackout by the RIS-aided network is NOMA. *U*_2_’s far-user performance is better than *U*_1_’s near-user for each of the same number of RIS elements. This is because, according to the author, the signal received by the distant-users *U*_2_ is a superposition of the signal received via RIS and the relay signal that was successfully encoded by *U*_1_.

Next, the use of the RIS-aided NOMA network could reduce the presence of resources by eliminating amplification and forward (AF) modes and only using decode and forward (DF) modes, which is a weakness of the NOMA network if implemented massively. Thus, it can reduce the cost of its implementation.

This shows that the performance of the proposed RIS-NOMA system is efficient, as expected from our study.

The amount of bit errors is the total amount of bits that have been corrupted by interference, tampering, distortion, or bit synchronization issues after being received from the data stream across the communication channel. The goal rate parameter in Equations (9) and (10), which stands for the amount of bit errors per channel usage, is used by the authors to derive the expression that comes the closest to the blackout probability. The number of bit errors per channel is referred to as the target rate, and we utilize it as a constant with a specific value to choose the SNR threshold value. However, it is clear from Equations (9) and (10) that we employ that the bit error value increases as the SNR threshold value increases.

**Figure 5:**
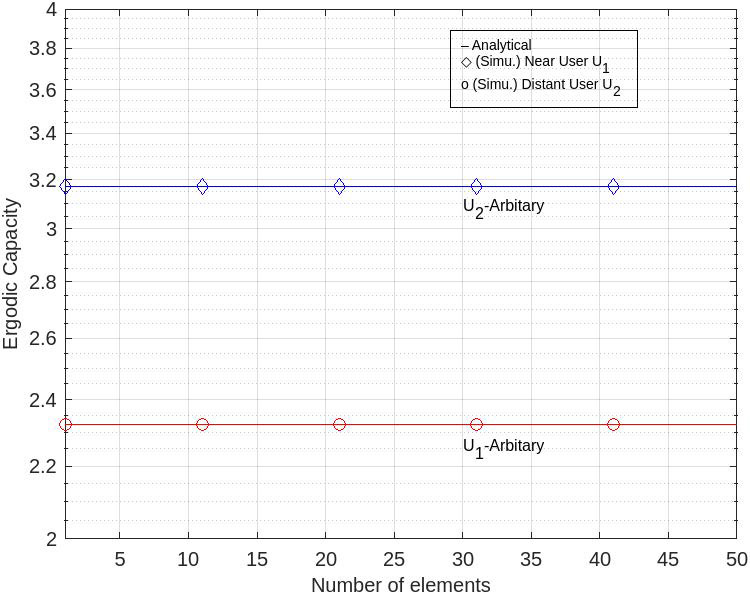
EC for arbitrary level at *U*_1_ and *U*_2_.

Figure 5 shows the profile of the ergodic capacity (EC) at *U*_1_ and *U*_2_ for arbitrary phase shifts. Here, there is no gap in EC as long as there is the addition of the number of RIS elements, both on *U*_1_ and *U*_2_. It is also shown that, for arbitrary phase shifts, the ergodic capacity value of distant-users *U*_2_ is higher than the ergodic capacity of near-users *U*_1_.

**Figure 6:**
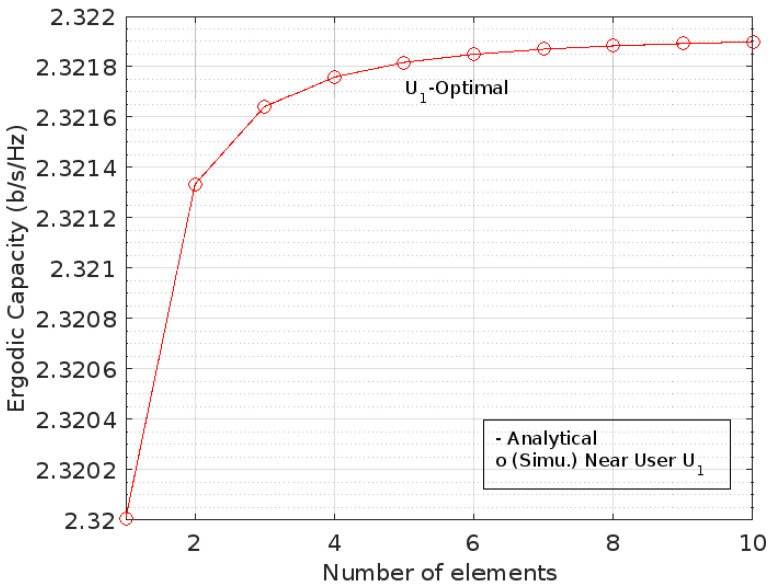
EC for optimal phase level at *U*_1_.

**Figure 7:**
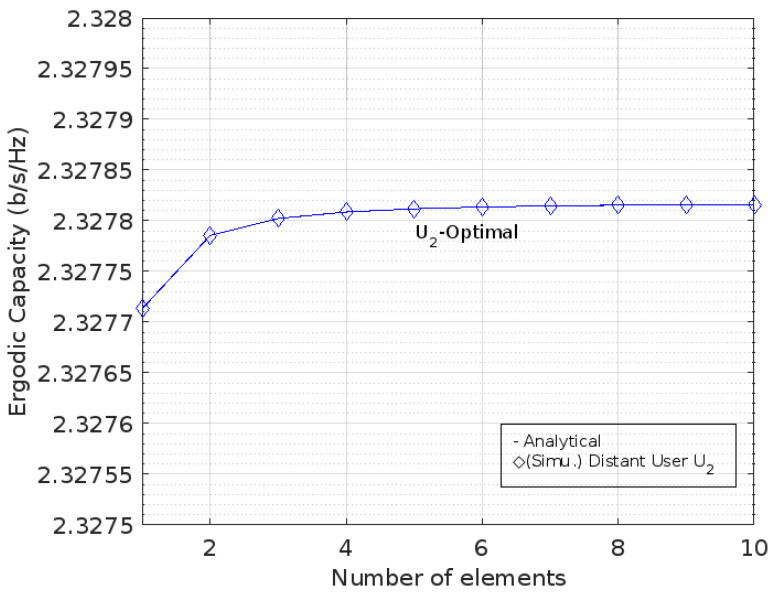
EC for optimal phase level at *U*_2_.

Figure 6 shows that the value of EC has a logarithmic trend, particularly in the range [0, 10] of element numbers for near user *U*_1_ with no gap after it. Meanwhile, Figure 7 also shows that EC has a logarithmic trend against the addition of RIS elements on distant user *U*_2_. 

**Figure 8:**
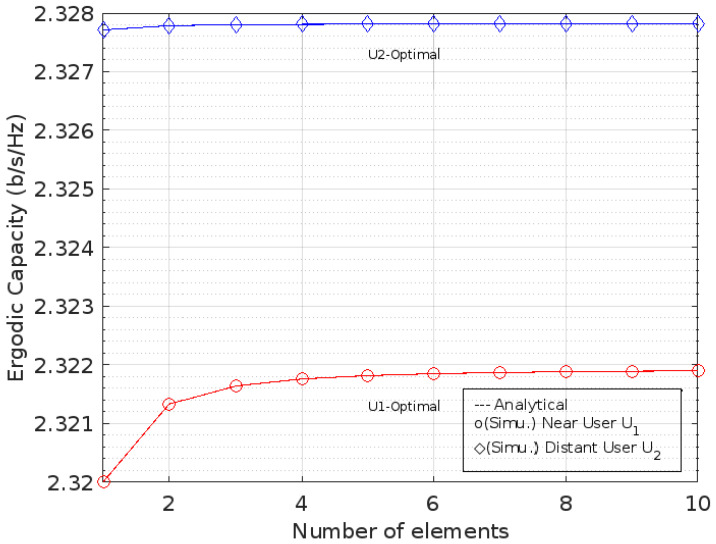
Comparison of EC for optimal phase level at *U*_1_ and *U*_2_.

This is different from the optimal phase shift condition. In Figure 6 and Figure 7, both of the ECs for the optimal phase shifts against the addition of the number of RIS elements on *U*_1_ and *U*_2_, respectively, are shown, whereas Figure 8 describes the comparison between them.

According to the author, the absence of a gap between the ergodic capacity values and the addition of the number of RIS elements in Figure 5 and Figure 7 is caused by the calculation process. There is no visible gap in EC as long as the number of RIS elements is increased. However, the ergodic capacity has slightly different values by adding the number of RIS elements.

Moreover, there is the effect of mutual interference. As it is known that, in deriving the blackout probability equation, we use interference parameters which are affected by RIS elements, attenuation is affected by path-loss distance and noise. Because the analyzed network is based on NOMA, the most decisive thing is the user’s proximity to the base operation, which is when, using NOMA, based on the authors’ analysis, the probability of a blackout becomes bigger, whereas, when using NOMA assisted by RIS, there is almost no big difference in probability to the distance of the closest user to the base operation. Meanwhile, the greater the number of RIS elements, the smaller the blackout probability value.

The assumption of near users and far users is actually a zoning, where the signal transmitted via reflection from the RIS depends on changes in the angle of reflection of the RIS elements that affect the phase shift. Therefore, the speed of the user affects the reception of the signal by the user. Because the limit of the change in the angle of reflection is between 0 and180, the faster the user passes through the zones of the RIS, the user will lose the signal (Blank-spot). Based on this, it is necessary to install another RIS antenna.

## 7. Conclusions

This paper investigates the RIS-aided downlink in an NOMA network with perfect-channel statistics information (*p*-CSI) over Nakagami-*m* fading channels without direct link scenarios. During outages, the performance of cooperative relaying scenarios is examined in depth. By using an incomplete gamma distribution and statistical information about each channel, we derive the closed-form expression of the coverage probability for the RIS-aided downlink in an NOMA network for enhanced communication with arbitrary phase shifts. The closed-form expression of the coverage probability is constructed to characterize the network’s outage behavior. Besides that, the closed-form expressions for ergodic capacity (upper bound of channel capacity) for arbitrary and optimal phase shifts were created. According to simulation data without a direct link from BO, the RIS-aided NOMA has a lower outage probability than the traditional NOMA. All the obtained closed-form expressions coincide with Monte Carlo simulations and show that the coverage probability of the distant user outperforms the nearby user. However, the greater the number of RIS elements, the wider the coverage probability. Furthermore, based on the asymptotic analysis and the upper bound on channel capacity, they reveal the scaling law of the number of phase shifts at the RIS-aided NOMA. The ergodic capacity of the distant user outperforms the near user, both in arbitrary and optimal phase shifts. In the future, we will drop the half-duplex assumption in favor of investigating the impact of loop interference on system performance and ergodic capacity when imperfect channel statistic information (*Ip*-CSI) is used.

## Figures and Tables

**Table 1:** Notations.

Notation	Definition
y(t)	superpose of the signal that is targeted to Near User (*U*_1_) and Far User (*U*_2_)
$P_{s}$	the normalized power of a transmitted signal
$P_{1}$	the normalized power of the transmission signal at *U*_1_
$a_{1}$ $\;\mathrm{and}\;a_{2}$	$\mathrm{the}\;\mathrm{level}\;\mathrm{power}\;\mathrm{of}\;\mathrm{the}\;\mathrm{signal}\;x_{1}$$\;\mathrm{and}\;x_{2}$, respectively
L	number of RIS elements
α	the amplitude reflection coefficient with α∈(0,1]
$\theta_{l}$	the adjustable phase applied by the l-th reflecting element of RIS
Φ	$\mathrm{The}\;\mathrm{phase}-\mathrm{shift}\;\mathrm{matrix},\;\mathrm{diag}(\exp(j\theta_{1}$$),\;\exp(j\theta_{2}$$),\;\ldots ,\;\exp(j\theta_{L}$)
${\left(.\right)}^{H}$	Hermitian transpose
$\beta_{k}$	the large-scale fading coefficients of channel *k*
$\Omega_{k}$	the link power of channel *k*
${\hat{\Omega }}_{k}$	the average connection power of channel *k*
${\hat{h}}_{k}$	average fading coefficient of channel *k*
$h_{k}$	the fading coefficient of channel *k*
${\hat{X}}_{\upsilon }$	average gain of fading coefficient by RIS for the υ user
$X_{\upsilon }$	gain of fading coefficient by RIS for the υ user
$e_{k}$	channel estimation error
$\sigma_{e_{k}}^{2}$	variant of channel estimation error
$\eta_{k}$	relative channel estimation error of channel *k*
$m_{\upsilon }$	the shape factor of the gamma distribution of the channel at υ
χ	path-loss exponent
$d_{k}$	the distance of two points crossed by the channel *k*
$P_{\left(..\right)}$	outage probability at (..)
$n_{U_{1}}$ $\;\mathrm{and}\;n_{U_{2}}$	the AWGN at *U*_1_ and *U*_2_, respectively
$d_{SU_{1}},d_{SR_{1}},d_{RU_{1}}$	$\mathrm{the}\;\mathrm{distances}\;\mathrm{for}\;BS-U_{1},\;BS-RIS,\;and\;RIS-U_{1}$, respectively
$d_{SU_{2}},d_{SR_{2}},d_{RU_{2}}$	$\mathrm{the}\;\mathrm{distances}\;\mathrm{for}\;BS-U_{2},\;BS-RIS,\;and\;RIS-U_{2}$, respectively
$h_{D_{1}},h_{D_{2}},h_{l},g_{1l},g_{2l}$	the coefficients of fading channels
$\rho_{s}$	the transmit signal to noise ratio (SNR)
$\rho_{x_{2}\to x_{1}}$	the received signal to interference and noise ratio (SINR) for *U*_1_ to decode signal *x*_2_ of *U*_2_
$\rho_{U_{1}},\rho_{U_{2}}$	the received SINR for *U*_1_ and *U*_2_ to decode its own signal, respectively
$\rho_{2,U2}$	the received SINR for *U*_2_ to decode signal *x*_2_ for relaying link
$\rho_{U_{2}}^{SC}$	the received SINR after selection combining (*SC*) at *U*_2_
$\rho_{Th_{1}}$ $\;\mathrm{and}\;\rho_{Th_{2}}$	target SINR of user *u*_1_ and *u*_2_, respectively
$\zeta_{1}$ $\;\mathrm{and}\;\zeta_{2}$	target rate of user *u*_1_ and *u*_2_, respectively
$P_{U_{1}}$ $\;\mathrm{and}\;P_{U_{2}}$	outage probability at *U*_1_ and *U*_2_
$\tau_{1}$ $,\;\tau_{2}$ $\;\mathrm{and}\;\tau_{3}$	as the *first*-*comparison –parameter,* the *second*-*comparison-parameter and* the *third*-*comparison -parameter*
$\lambda_{1}$ $,\;\lambda_{2}$	interference and noise due to the using of RIS-aided at *U*_1_ and *U*_2_ respectively
$\delta_{1}$ $,\;\delta_{2}$	the scale factor of the gamma distribution of the channel at **U*_1_ and U*_2_ respectively

**Table 2:** The parameters for the simulations in this paper.

Notation	Definition
Power allocation coefficient	$a_{1}=0.2$, $a_{2}=0.8$
Path loss exponent	$\chi_{k}=2;$ $\;k\in \left\{SU_{1},SR_{1},RU_{1},SU_{2},SR_{2},RU_{2},U_{1}U_{2}\right\}$
Distance between two nodes	$\begin{array}{l} d_{SU_{1}}=0.05(50m),d_{SR_{1}}=d_{SR_{2}}=0.06(60m),d_{RU_{1}}=1,d_{SU_{2}}=0.04\sim 1,\\ d_{RU_{2}}=0.04\sim 1,d_{UU_{2}}=1-d_{SU_{1}} \end{array}$
Relative channel estimation error	$\eta_{k}=1\times 10^{-4}\sim 9\times 10^{-4};k\in \left\{SU_{1},SR_{1},RU_{1},SU_{2},SR_{2},RU_{2},U_{1}U_{2}\right\}$
Transmit SNR	$\rho_{s}=0\sim 60dB$
Transmitter and Receiver Gain	$G_{t}=3.2dB_{i},G_{r}=1.3dB_{i}$
Target Rate	$\zeta_{1}=3.6BPCU,\zeta_{2}=1BPCU$

## Appendix A. Proof of proposition—$U_{1}$

It is defined that $X_{v}={\left|\sum_{l=1}^{N}h_{vl}^{H}\Phi g_{vl}\right|}^{2}$ if it is assumed that the reflected signal by RIS elements have arbitrary phase shifts. Due to each channel being identical independent distribution (*i.i.d*), then it could be assumed ${\hat{h}}_{l}\sim \mathbb{C}\mathbb{N}\left(0,\beta_{SR}I_{N}\right)$,${\hat{g}}_{vl}\sim \mathbb{C}\mathbb{N}\left(0,\beta_{RU_{v}}I_{N}\right)$, and $\phi =diag\left(e^{j\theta_{1}},e^{j\theta_{2}},\ldots ,e^{j\theta_{N}}\right)$, where $I_{N}$ and $\theta_{n}\in \left[-\pi ,\pi \right]$ are identity matrices with order N with range of $\theta_{n}$, respectively.

Furthermore, we define the expected value $E\left\{\left|X_{v}\right|\right\}$ is the mean of the independence of the channels that compose it. It could be expressed by Equation (A1).

(A1) $$E\left\{\left|X_{v}\right|\right\}=E\left\{{\left|h_{l}^{H}\Phi g_{vl}\right|}^{2}\right\}$$

$X_{v}$ is the summation between average of link ${\hat{X}}_{v}$ and fading caused by path-loss-distance attenuation $e_{v}{}^{2}$ in a propagation environment and could be expressed as Equation (A2).

(A2) $$E\left\{\left|X_{v}\right|\right\}=E\left\{{\hat{X}}_{v}+\sigma_{e_{v}}^{2}\right\}=E\left\{{\hat{X}}_{v}\right\}+\sigma_{e_{v}}^{2}$$

The attenuation caused by path-loss distance $e_{v}{}^{2}$ could be defined as $\frac{\eta_{SR}}{N}\left(\;d_{SR}^{-\chi }\right).\frac{\eta_{RU_{v}}}{N}\left(\;d_{RU_{v}}^{-\chi }\right)$; then Equation (A2) could be rewritten as Equation (A3).

(A3) $$E\left\{\left|X_{v}\right|\right\}=E\left\{{\left|{\hat{h}}_{l}^{H}\Phi {\hat{g}}_{vl}\right|}^{2}\right\}+\frac{\eta_{SR}}{N}\left(d_{SR}^{?\chi }\right)\cdot \frac{\eta_{RU_{v}}}{N}\left(d_{RU_{v}}^{?\chi }\right)$$

We define that $E\left\{{\left|{\hat{h}}_{l}^{H}\Phi {\hat{g}}_{vl}\right|}^{2}\right\}=N\beta_{SR}\beta_{RU_{v}}$ so Equation (A3) can be written as Equation (A4):

(A4) $$E\left\{\left|X_{v}\right|\right\}=N\beta_{SR}\beta_{RU_{v}}+\frac{\eta_{SR}}{N}\left(\;d_{SR}^{-\chi }\right).\frac{\eta_{RU_{v}}}{N}\left(\;d_{RU_{v}}^{-\chi }\right)$$

Furthermore, $E\left\{{\left|{\hat{X}}_{v}\right|}^{2}\right\}$ is determined as Equation (A5) below.

(A5) $$E\left\{{\left|{\hat{X}}_{v}\right|}^{2}\right\}=E\left\{{\left|{\hat{h}}_{l}^{H}\Phi {\hat{g}}_{vl}\right|}^{2}\right\}$$

According to the circular symmetric properties, Equation (A5) could be written as Equation (A6):

(A6) $$E\left\{{\left|{\hat{X}}_{v}\right|}^{2}\right\}=E\left\{{\left|\left|\Phi {\hat{g}}_{vl}\right|\right|}^{4}{\left|\frac{{\hat{h}}_{l}^{H}\Phi {\hat{g}}_{vl}}{\left|\left|\Phi {\hat{g}}_{vl}\right|\right|}\frac{{\hat{g}}_{vl}^{H}\Phi^{H}{\hat{h}}_{l}}{\left|\left|\Phi {\hat{g}}_{vl}\right|\right|}\right|}^{2}\right\}$$

If it is defined $z=\frac{{\hat{h}}_{l}^{H}\Phi {\hat{g}}_{vl}}{\left|\left|\Phi {\hat{g}}_{vl}\right|\right|};z\sim CN(0,\beta_{SR})$, then Equation (A6) could be expressed as Equation (A7).

(A7) $$E\left\{{\left|{\hat{X}}_{v}\right|}^{2}\right\}=E\left\{{\left|\left|\Phi {\hat{g}}_{vl}\right|\right|}^{4}{\left|z\right|}^{4}\right\}=E\left\{{\left|\left|\Phi {\hat{g}}_{vl}\right|\right|}^{4}\right\}E\left\{{\left|z\right|}^{4}\right\}$$

Equation (A8) is obtained by substituting the values of $E\left\{{\left|\left|\Phi {\hat{g}}_{vl}\right|\right|}^{4}\right\}E\left\{{\left|z\right|}^{4}\right\}$. Next, it is also defined that $X_{v}={\hat{X}}_{v}+e_{v}={\hat{X}}_{v}+\sigma_{e_{v}}^{2}$. Therefore, the expected value of $X_{v}{}^{2}$ could be stated in Equation (A8).

(A8) $$E\left\{{\left|{\hat{X}}_{v}\right|}^{2}\right\}=N(N+1)\beta_{RU_{v}}^{2}2\beta_{SR}^{2}=(2N^{2}+2N)\beta_{RU_{v)}}^{2}\beta_{SR}^{2}$$

Next, we define $E\left\{{\left|X_{v}\right|}^{2}\right\}$=$E\left\{{\left|{\hat{X}}_{v}\right|}^{2}\right\}+\sigma_{e_{v}}^{2}$, where $\sigma_{e_{v}}^{2}=\left|\frac{\eta_{SR}}{N}\left(\;d_{SR}^{-\chi }\right).\frac{\eta_{RU_{v}}}{N}\left(\;d_{RU_{v}}^{-\chi }\right)\right|$ the according to Equation (A8), we could be rewritten as Equations (A9) and (A10).

(A9) $$E\left\{{\left|X_{v}\right|}^{2}\right\}=E\left\{{||{\hat{h}}_{l}^{H}\Phi {\hat{g}}_{vl}|}^{2}+|\frac{\eta_{SR}}{N}\left(d_{SR}^{-\chi }\right)\cdot \frac{\eta_{RU_{v}}}{N}\left(d_{RU_{v}}^{-\chi }\right){∥}^{2}\right\}$$

(A10) $$E\left\{{\left|X_{v}\right|}^{2}\right\}=(2N^{2}+2N)\beta_{RU_{v}}^{2}\beta_{SR}^{2}+{\left|\frac{\eta_{SR}}{N}\left(\;d_{SR}^{-\chi }\right).\frac{\eta_{RU_{v}}}{N}\left(\;d_{RU_{v}}^{-\chi }\right)\right|}^{2}$$

By defining $Var\left\{\left|X_{v}\right|\right\}=E\left\{{\left|X_{v}\right|}^{2}\right\}-{\left|E\left\{\left|X_{v}\right|\right\}\right|}^{2}$, then it could be expressed as Equation (A11). Thus, we obtain the equation of *m*_1_ in Equation (A12) by using Equation (15).

(A11) $$Var\left\{\left|X_{v}\right|\right\}=(N^{2}+2N)\beta_{RU_{v}}^{2}\beta_{SR}^{2}-2N\beta_{SR}\beta_{RU_{v}}\frac{\eta_{SR}}{N}\left(\;d_{SR}^{-\chi }\right).\frac{\eta_{RU_{v}}}{N}\left(\;d_{RU_{v}}^{-\chi }\right)$$

(A12) $$m_{1}=\frac{(2N^{2}+2N)\beta_{RU_{1}}^{2}\beta_{SR}^{2}+{\left|\frac{\eta_{SR}}{N}\left(\;d_{SR}^{-\chi }\right).\frac{\eta_{RU_{v}}}{N}\left(\;d_{RU_{1}}^{-\chi }\right)\right|}^{2}}{(N^{2}+2N)\beta_{RU_{1}}^{2}\beta_{SR}^{2}-2N\beta_{SR}\beta_{RU_{1}}\frac{\eta_{SR}}{N}\left(\;d_{SR}^{-\chi }\right).\frac{\eta_{RU_{v}}}{N}\left(\;d_{RU_{1}}^{-\chi }\right)}$$

In addition, $\tau_{1}$ in Equation (11) could be derived by defining $\rho_{Th_{1}}$ in Equation (A13).

(A13) $$\rho_{Th_{1}}=\frac{\left(\;2^{2\zeta_{1}}-1\right)}{\rho_{s}}$$

From Equation (7), we define $A={\left|\sum_{l=1}^{L}\left|{\hat{h}}_{l}\right|\left|{\hat{g}}_{1l}\right|\right|}^{2}$ and $\lambda_{1}=\frac{\eta_{SR}}{N}\left(\;d_{SR}^{-\chi }\right).\frac{\eta_{RU_{1}}}{N}\left(\;d_{RU_{1}}^{-\chi }\right)\rho_{s}+1$. Next, Equation (7) could be rewritten as Equation (A19) via the derived steps shown in Equations (A14)–(A18).

(A14) $$\frac{Aa_{1}\rho_{s}}{Aa_{1}\rho_{s}+\lambda_{1}}\geq \rho_{Th_{1}}$$

(A15) $$\Rightarrow Aa_{1}\rho_{s}\geq \left(Aa_{1}\rho_{s}+\lambda_{1}\right)\rho_{Th_{1}}$$

(A16) $$\Rightarrow Aa_{1}\rho_{s}-Aa_{1}\rho_{s}\rho_{Th_{1}}\geq \lambda_{1}\rho_{Th_{1}}$$

(A17) $$\Rightarrow A\rho_{s}\left(a_{1}-a_{1}\rho_{Th_{1}}\right)\geq \lambda_{1}\rho_{Th_{1}}$$

(A18) $$\Rightarrow A\geq \frac{\lambda_{1}\rho_{Th_{1}}}{\left(a_{1}-a_{1}\rho_{Th_{1}}\right)\rho_{s}}$$

(A19) $$\Rightarrow \tau_{1}=\frac{\lambda_{1}\rho_{Th_{1}}}{\left(a_{1}-a_{1}\rho_{Th_{1}}\right)\rho_{s}}$$

Similarly, $\rho_{Th_{2}}$ is defined in Equation (A20):

(A20) $$\rho_{Th_{2}}=\frac{\left(\;2^{2\zeta_{2}}-1\right)}{\rho_{s}}$$

Then, $\tau_{2}$ could be derived from Equation (A26) via the derived steps shown in Equations (A21)–(A25) as follows.

(A21) $$\tau_{2}=\frac{Aa_{2}\rho_{s}}{Aa_{1}\rho_{s}+\lambda_{1}}\geq \rho_{Th_{2}}$$

(A22) $$\Rightarrow Aa_{2}\rho_{s}\geq \left(Aa_{1}\rho_{s}+\lambda_{1}\right)\rho_{Th_{2}}$$

(A23) $$\Rightarrow Aa_{2}\rho_{s}-Aa_{1}\rho_{s}\rho_{Th_{2}}\geq \lambda_{1}\rho_{Th_{2}}$$

(A24) $$\Rightarrow A\rho_{s}\left(a_{2}-a_{1}\rho_{Th_{2}}\right)\geq \lambda_{1}\rho_{Th_{2}}$$

(A25) $$\Rightarrow A\geq \frac{\lambda_{1}\rho_{Th_{2}}}{\left(a_{2}-a_{1}\rho_{Th_{2}}\right)\rho_{s}}$$

(A26) $$\Rightarrow \tau_{2}=\frac{\lambda_{1}\rho_{Th_{2}}}{\left(a_{2}-a_{1}\rho_{Th_{2}}\right)\rho_{s}}$$

By using the Nakagami-*m* distribution for the fading channel $\hat{h}$, then the probability density function (PDF) could be expressed as $f_{{\left|\hat{h}\right|}^{2}}\left(x\right)=\frac{m^{m}d^{xm}x^{m-1}}{{\left(1-\eta \right)}^{m}\left(m-1\right)!}e^{-\frac{md^{x}x}{1-\eta }}$ and the cumulative distributive function (CDF) could be expressed as $F_{{\left|\hat{h}\right|}^{2}}\left(x\right)=1-e^{-\frac{md^{x}x}{1-\eta }}\sum_{i-0}^{m-1}\frac{1}{i!}{\left(\frac{md^{x}x}{1-\eta }\right)}^{i}$.

Next, by modifying the equations of PDF and CDF mentioned above caused by RIS implementation in the NOMA networks, then Equations (A27) and (A28) are obtained and shown below.

(A27) $$f_{{\left|{\hat{X}}_{v}\right|}^{2}}\left(\tau \right)={\left(\delta_{v}\right)}^{m_{v}}\frac{\tau^{m_{v}-1}}{\left(m_{v}-1\right)!}e^{-\delta_{v}\tau }$$

(A28) $$F_{{\left|{\hat{X}}_{v}\right|}^{2}}\left(\tau \right)=1-e^{-\delta_{v}\tau }\sum_{i-0}^{m_{v}-1}\frac{1}{i!}{\left(\delta_{v}\tau \right)}^{i}$$

where v = 1 for the $\delta_{1}$ in Equation (22).

Next, it is defined as Equation (A29).

(A29) $$P_{U_{1}}=1-\mathrm{Pr}\left({\left|{\hat{X}}_{1}\right|}^{2}>\tau_{2},{\left|{\hat{X}}_{1}\right|}^{2}>\tau_{1}\right)$$

Finally, Equations (A30) and (A31) is obtained by rearranging Equation (A29).

(A30) $$P_{U_{1}}=1-\mathrm{Pr}\left({\left|{\hat{X}}_{1}\right|}^{2}>\max\left(\tau_{2},\tau_{1}\right)\right)$$

(A31) $$P_{U_{1}}=1-\left({\left|{\hat{X}}_{1}\right|}^{2}>\tau \right)$$

where τ could be $\tau_{2}$ or $\tau_{1}$ dependent on τ, which has the largest value. This is conducted based on consideration of the probability that *U*_1_ fails to decode the signal *x*_2_ from distant user *U*_2_ and/or its own signal *x*_2_. Thus, we use the larger probability failure from those two events. Therefore, Equation (A31) is stated as Equation (A32).

(A32) $$P_{U_{1}}=1-e^{-\delta_{1}\tau }\sum_{j-0}^{m_{1}-1}\frac{{\left(\delta_{1}\tau \right)}^{j}}{j!}$$

□

## Appendix B. Proof of proposition—$U_{2}$

Similar to the steps in Appendix A, *τ*_3_ could be derived from Equation (24). It is defined that $B={\left|\sum_{l=1}^{L}\left|{\hat{h}}_{l}\right|\left|{\hat{g}}_{2l}\right|\right|}^{2}$ and $\lambda_{2}=\frac{\eta_{SR}}{N}\left(\;d_{SR}^{-\chi }\right).\frac{\eta_{RU_{1}}}{N}\left(\;d_{RU_{2}}^{-\chi }\right)\rho_{s}+1$. According to the Equations (A33)–(A37) below, we could derive $\tau_{3}$ shown in Equation (A38).

(A33) $$\tau_{3}=\frac{Ba_{2}\rho_{s}}{Ba_{1}\rho_{s}+\lambda_{2}}\geq \rho_{Th_{2}}$$

(A34) $$\Rightarrow Ba_{2}\rho_{s}\geq (Ba_{1}\rho_{s}+\lambda_{2})\rho_{Th_{2}}$$

(A35) $$\Rightarrow Ba_{2}\rho_{s}-Ba_{1}\rho_{s}\rho_{Th_{2}}\geq \lambda_{2}\rho_{Th_{2}}$$

(A36) $$\Rightarrow B\rho_{s}\left(a_{2}-a_{1}\rho_{Th_{2}}\right)\geq \lambda_{2}\rho_{Th_{2}}$$

(A37) $$\Rightarrow B\geq \frac{\lambda_{2}\rho_{Th_{2}}}{\left(a_{2}-a_{1}\rho_{Th_{2}}\right)\rho_{s}}$$

(A38) $$\Rightarrow \tau_{3}=\frac{\lambda_{2}\rho_{Th_{2}}}{\left(a_{2}-a_{1}\rho_{Th_{2}}\right)\rho_{s}}$$

Furthermore, *m*_2_ could be obtained by using Equation (A39) shown below.

(A39) $$m_{2}=\frac{(2N^{2}+2N)\beta_{RU_{2}}^{2}\beta_{SR}^{2}+{\left|\frac{\eta_{SR}}{N}\left(\;d_{SR}^{-\chi }\right).\frac{\eta_{RU_{2}}}{N}\left(\;d_{RU_{2}}^{-\chi }\right)\right|}^{2}}{(N^{2}+2N)\beta_{RU_{2}}^{2}\beta_{SR}^{2}-2N\beta_{SR}\beta_{RU_{2}}\frac{\eta_{SR}}{N}\left(\;d_{SR}^{-\chi }\right).\frac{\eta_{RU_{2}}}{N}\left(\;d_{RU_{2}}^{-\chi }\right)}$$

According to Equation (19), the following Equations (A40)–(A45) could be derived:

(A40) $$P_{U_{2}}=\mathrm{Pr}\left(\rho {(}_{U_{1,}x_{2}\to x_{1}})<\rho_{Th_{2}}\right)+\mathrm{Pr}\left(\rho_{2,U_{2}}<\rho_{Th_{2}},\rho {(}_{U_{1,}x_{2}\to x_{1}})>\rho_{Th_{2}}\right)$$

(A41) $$P_{U_{2}}=\mathrm{Pr}\left({\left|X_{1}\right|}^{2}<\tau_{2}\right)+\mathrm{Pr}\left({\left|X_{2}\right|}^{2}<\tau_{3},{\left|X_{1}\right|}^{2}>\tau_{2}\right)$$

(A42) $$P_{U_{2}}=\mathrm{Pr}\left({\left|X_{1}\right|}^{2}<\tau_{2}\right)+\mathrm{Pr}\left({\left|X_{1}\right|}^{2}>\tau_{2}\right).\mathrm{Pr}\left({\left|X_{2}\right|}^{2}<\tau_{3}\right)$$

(A43) $$P_{U_{2}}=1-\mathrm{Pr}\left({\left|X_{1}\right|}^{2}>\tau_{2}\right)+\mathrm{Pr}\left({\left|X_{1}\right|}^{2}>\tau_{2}\right).\mathrm{Pr}\left({\left|X_{2}\right|}^{2}<\tau_{3}\right)$$

(A44) $$P_{U_{2}}=1-\mathrm{Pr}\left({\left|X_{1}\right|}^{2}>\tau_{2}\right)\left(1-\mathrm{Pr}\left({\left|X_{2}\right|}^{2}<\tau_{3}\right)\right)$$

(A45) $$P_{U_{2}}=1-\mathrm{Pr}\left({\left|X_{1}\right|}^{2}>\tau_{2}\right).\mathrm{Pr}\left({\left|X_{2}\right|}^{2}>\tau_{3}\right)$$

Finally, they could be obtained by using Equations (A46) and (A47).

(A46) $$P_{U_{2}}=1-e^{-\delta_{1}\tau_{2}}\sum_{j-0}^{m_{1}-1}\frac{{\left(\delta_{1}\tau_{2}\right)}^{j}}{j!}.e^{-\delta_{2}\tau_{3}}\sum_{k-0}^{m_{2}-1}\frac{{\left(\delta_{2}\tau_{3}\right)}^{k}}{k!}$$

(A47) $$P_{U_{2}}=1-e^{-\left(\delta_{1}\tau_{2}+\delta_{2}\tau_{3}\right)}\sum_{j=0}^{m_{1}-1}\sum_{k=0}^{m_{2}-1}\frac{{\left(\delta_{1}\tau_{2}\right)}^{j}{\left(\delta_{2}\tau_{3}\right)}^{k}}{j!k!}$$

□

## Appendix C. Proof of proposition—$\tau_{2}$

Equation (32) gives the general form of the instantaneous channel capacity (b/s/Hz) when assuming a coherent combination. By assuming the phase shifts of RIS’s elements are arbitrary, then we could define $X_{1}=|h_{1l}^{H}\phi g_{1l}|$. Furthermore, the arbitrary level of ergodic capacity for *U*_1_ is mentioned Equation (A48) as follows:

(A48) $${\hat{C}}_{U_{1}Abtr}=E\left\{\log_{2}\left(1+\rho {(}_{U_{1,}x_{2}\to x_{1}}\right)\right\}$$

We use $\rho {(}_{U_{1,}x_{2}\to x_{1}})$ as the SINR at $U_{1}$, Thus, Equation (7) is obtained. After rearranging, Equation (A49) is obtained. In addition, we could rewrite Equation (A49) as Equation (A50) by using the proposition in Appendix A. By assuming the phase shifts of RIS’s elements are optimal, then we could define $X_{1}=\left|\sum_{l=1}^{N}\left|{\hat{h}}_{1}l\right|\left|{\hat{g}}_{1l}\right|\right|$. Next, both ${\hat{C}}_{U_{1}Abtr}$ and ${\hat{C}}_{U_{1}Abtr}$ could be obtained in Equations (A49) and (A50).

(A49) $${\hat{C}}_{U_{1}Abtr}=\log_{2}\left(1+E\left\{\frac{\left({\left|{\hat{h}}_{1}l^{H}\Phi {\hat{g}}_{1l}\right|}^{2}\right)a_{2}\rho_{s}}{\left({\left|{\hat{h}}_{1}l^{H}\Phi {\hat{g}}_{1l}\right|}^{2}\right)a_{1}\rho_{s}+\left(\frac{\eta_{SR}}{N}\left(\;d_{SR}^{-\chi }\right).\frac{\eta_{RU_{1}}}{N}\left(\;d_{RU_{1}}^{-\chi }\right)\right)\rho_{s}+1}\right\}\right)$$

(A50) $${\hat{C}}_{U_{1}Abtr}=\log_{2}\left(1+\frac{E\left\{\left({\left|{\hat{h}}_{1}l^{H}\Phi {\hat{g}}_{1l}\right|}^{2}\right)\right\}a_{2}\rho_{s}}{E\left\{\left({\left|{\hat{h}}_{1}l^{H}\Phi {\hat{g}}_{1l}\right|}^{2}\right)\right\}a_{1}\rho_{s}+\left(\frac{\eta_{SR}}{N}\left(\;d_{SR}^{-\chi }\right).\frac{\eta_{RU_{1}}}{N}\left(\;d_{RU_{1}}^{-\chi }\right)\right)\rho_{s}+1}\right)$$

According to [15], for the optimal phase shifts, the $E\left\{X_{1}\right\}$ could be determined by using Equation (A51) shown below.

(A51) $$E\left\{\left|\sum_{l=1}^{N}\left|{\hat{h}}_{l}\right|\left|{\hat{g}}_{1l}\right|\right|\right\}=\frac{N\pi \sqrt{\beta_{SR}\beta_{RU_{1}}}}{4}\;$$

and has variance in Equation (A52).

(A52) $$Var\left\{\left|\sum_{l=1}^{N}\left|{\hat{h}}_{l}\right|\left|{\hat{g}}_{1l}\right|\right|\right\}=N\left(1-\frac{\pi^{2}}{16}\right)\beta_{SR}\beta_{RU_{1}}$$

According to Equation (A52) and Equation (A51), then $E\left\{{\left|\sum_{l=1}^{N}\left|{\hat{h}}_{l}\right|\left|{\hat{g}}_{1l}\right|\right|}^{2}\right\}$ could be obtained by using the derived steps from Equation (A53) to Equation (A55). Finally, the optimal level of ergodic capacity for *U*_1_ is mentioned as Equations (A56)–(A58).

(A53) $$E\left\{{\left|\sum_{l=1}^{N}\left|{\hat{h}}_{l}\right|\left|{\hat{g}}_{1l}\right|\right|}^{2}\right\}=Var\left\{\left|\sum_{l=1}^{N}\left|{\hat{h}}_{l}\right|\left|{\hat{g}}_{1l}\right|\right|\right\}+{\left(E\left\{\left|\sum_{l=1}^{N}\left|{\hat{h}}_{l}\right|\left|{\hat{g}}_{1l}\right|\right|\right\}\right)}^{2}$$

(A54) $$E\left\{{\left|\sum_{l=1}^{N}\left|{\hat{h}}_{l}\right|\left|{\hat{g}}_{1l}\right|\right|}^{2}\right\}=N\left(1-\frac{\pi^{2}}{16}\right)\beta_{SR}\beta_{RU1}+{\left(\frac{N\pi \sqrt{\beta_{SR}\beta_{RU_{1}}}}{4}\right)}^{2}$$

(A55) $$E\left\{{\left|\sum_{l=1}^{N}\left|{\hat{h}}_{l}\right|\left|{\hat{g}}_{1l}\right|\right|}^{2}\right\}=\left(\left(N\left(1-\frac{\pi^{2}}{16}\right)\right)\beta_{RU_{1}}\beta_{SR}+\frac{N^{2}\pi^{2}}{16}\beta_{RU_{1}}\beta_{SR}\right)$$

(A56) $${\hat{C}}_{U_{1}Op}=E\left\{\log_{2}\left(1+\frac{\left({\left|\sum_{l=1}^{N}\left|{\hat{h}}_{l}\right|\left|{\hat{g}}_{1l}\right|\right|}^{2}\right)a_{2}\rho_{s}}{\left({\left|\sum_{l=1}^{N}\left|{\hat{h}}_{1l}\right|\left|{\hat{g}}_{1l}\right|\right|}^{2}\right)a_{1}\rho_{s}+\lambda_{1}}\right)\right\}$$

(A57) $${\hat{C}}_{U_{1}Op}=E\left\{\log_{2}\left(1+\frac{\left({\left|\sum_{l=1}^{N}\left|{\hat{h}}_{l}\right|\left|{\hat{g}}_{1l}\right|\right|}^{2}\right)a_{2}\rho_{s}}{\left({\left|\sum_{l=1}^{N}\left|{\hat{h}}_{1l}\right|\left|{\hat{g}}_{1l}\right|\right|}^{2}\right)a_{1}\rho_{s}+\frac{\eta_{SR}}{N}\left(\;d_{SR}^{-\chi }\right).\frac{\eta_{RU_{1}}}{N}\left(\;d_{RU_{1}}^{-\chi }\right)\rho_{s}+1}\right)\right\}$$

(A58) $${\hat{C}}_{U_{1}Op}=\log_{2}\left(1+\frac{\left(\left(N\left(1-\frac{\pi^{2}}{16}\right)\right)\beta_{RU_{2}}\beta_{SR}+\frac{N^{2}\pi^{2}}{16}\beta_{RU_{2}}\beta_{SR}\right)a_{2}\rho_{s}}{\left(\left(N\left(1-\frac{\pi^{2}}{16}\right)\right)\beta_{RU_{1}}\beta_{SR}+\frac{N^{2}\pi^{2}}{16}\beta_{RU_{1}}\beta_{SR}\right)a_{1}\rho_{s}+\left(\frac{\eta_{SR}}{N}\left(\;d_{SR}^{-\chi }\right).\frac{\eta_{RU_{2}}}{N}\left(\;d_{RU_{1}}^{-\chi }\right)\right)\rho_{s}+1}\right)$$

□

## Appendix D

It is similar to the previous steps in Appendix C that the ergodic capacity in arbitrary phase shifts for *U*_2_ could be determined in Equations (A59)–(A61).

(A59) $${\hat{C}}_{U_{2}Abtr}=E\left\{\log_{2}\left(1+\frac{\left({\left|{\hat{h}}_{l}^{H}\Phi {\hat{g}}_{1l}\right|}^{2}\right)a_{2}\rho_{s}}{\left({\left|{\hat{h}}_{l}^{H}\Phi {\hat{g}}_{1l}\right|}^{2}\right)a_{1}\rho_{s}+\lambda_{1}}+\frac{\left({\left|{\hat{h}}_{l}^{H}\Phi {\hat{g}}_{2l}\right|}^{2}\right)a_{2}\rho_{s}}{\left({\left|{\hat{h}}_{l}^{H}\Phi {\hat{g}}_{2l}\right|}^{2}\right)a_{1}\rho_{s}+\lambda_{2}}\right)\right\}$$

(A60) $${\hat{C}}_{U_{2}Abtr}=\log_{2}\left(1+E\left\{\frac{\left({\left|{\hat{h}}_{l}^{H}\Phi {\hat{g}}_{1l}\right|}^{2}\right)a_{2}\rho_{s}}{\left({\left|{\hat{h}}_{l}^{H}\Phi {\hat{g}}_{1l}\right|}^{2}\right)a_{1}\rho_{s}+\lambda_{1}}+\frac{\left({\left|{\hat{h}}_{l}^{H}\Phi {\hat{g}}_{2l}\right|}^{2}\right)a_{2}\rho_{s}}{\left({\left|{\hat{h}}_{l}^{H}\Phi {\hat{g}}_{2l}\right|}^{2}\right)a_{1}\rho_{s}+\lambda_{2}}\right\}\right)$$

(A61) $${\hat{C}}_{U_{2}Abtr}=\log_{2}\left(1+\frac{a_{2}\rho_{s}\left(2N^{2}+2N\right)\beta_{RU_{1}}^{2}\beta_{SR}^{2}}{a_{1}\rho_{s}\left(2N^{2}+2N\right)\beta_{RU_{1}}^{2}\beta_{SR}^{2}+\lambda_{1}}+\frac{a_{2}\rho_{s}\left(2N^{2}+2N\right)\beta_{RU_{2}}^{2}\beta_{SR}^{2}}{a_{1}\rho_{s}\left(2N^{2}+2N\right)\beta_{RU_{2}}^{2}\beta_{SR}^{2}+\lambda_{2}}\right)$$

Finally, the ergodic capacity for *U*_2_ could be determined in Equations (A62)–(A64) for the optimal phase shifts by applying the previous steps in Appendix C.

(A62) $${\hat{C}}_{U_{2}Op}=E\left\{\log_{2}\left(1+\frac{\left({\left|\sum_{l=1}^{N}\left|{\hat{h}}_{l}\right|\left|{\hat{g}}_{1l}\right|\right|}^{2}\right)a_{2}\rho_{s}}{\left({\left|\sum_{l=1}^{N}\left|{\hat{h}}_{l}\right|\left|{\hat{g}}_{1l}\right|\right|}^{2}\right)a_{1}\rho_{s}+\lambda_{1}}+\frac{\left({\left|\sum_{l=1}^{N}\left|{\hat{h}}_{l}\right|\left|{\hat{g}}_{2l}\right|\right|}^{2}\right)a_{2}\rho_{s}}{\left({\left|\sum_{l=1}^{N}\left|{\hat{h}}_{l}\right|\left|{\hat{g}}_{2l}\right|\right|}^{2}\right)a_{1}\rho_{s}+\lambda_{2}}\right)\right\}$$

(A63) $${\hat{C}}_{U_{2}Op}=\log_{2}\left(1+E\left\{\frac{\left({\left|\sum_{l=1}^{N}\left|{\hat{h}}_{l}\right|\left|{\hat{g}}_{1l}\right|\right|}^{2}\right)a_{2}\rho_{s}}{\left({\left|\sum_{l=1}^{N}\left|{\hat{h}}_{l}\right|\left|{\hat{g}}_{1l}\right|\right|}^{2}\right)a_{1}\rho_{s}+\lambda_{1}}+\frac{\left({\left|\sum_{l=1}^{N}\left|{\hat{h}}_{l}\right|\left|{\hat{g}}_{2l}\right|\right|}^{2}\right)a_{2}\rho_{s}}{\left({\left|\sum_{l=1}^{N}\left|{\hat{h}}_{l}\right|\left|{\hat{g}}_{2l}\right|\right|}^{2}\right)a_{1}\rho_{s}+\lambda_{2}}\right\}\right)$$

(A64) $${\hat{C}}_{U_{2}Op}=\log_{2}\left(\begin{array}{l} 1+\frac{\left(\left(N\left(1-\frac{\pi^{2}}{16}\right)\right)\beta_{RU_{1}}\beta_{SR}+\frac{N^{2}\pi^{2}}{16}\beta_{RU_{1}}\beta_{SR}\right)a_{2}\rho_{s}}{\left(\left(N\left(1-\frac{\pi^{2}}{16}\right)\right)\beta_{RU_{1}}\beta_{SR}+\frac{N^{2}\pi^{2}}{16}\beta_{RU_{1}}\beta_{SR}\right)a_{1}\rho_{s}+\lambda_{1}}\\ +\frac{\left(\left(N\left(1-\frac{\pi^{2}}{16}\right)\right)\beta_{RU_{2}}\beta_{SR}+\frac{N^{2}\pi^{2}}{16}\beta_{RU_{2}}\beta_{SR}\right)a_{2}\rho_{s}}{\left(\left(N\left(1-\frac{\pi^{2}}{16}\right)\right)\beta_{RU_{2}}\beta_{SR}+\frac{N^{2}\pi^{2}}{16}\beta_{RU_{2}}\beta_{SR}\right)a_{1}\rho_{s}+\lambda_{2}} \end{array}\right)$$

□
